# Lateral palatal foramina are not widespread in Artiodactyla and imply baleen in extinct mysticetes

**DOI:** 10.1038/s41598-024-60673-8

**Published:** 2024-05-03

**Authors:** Eric G. Ekdale, Joseph J. El Adli, Michael R. McGowen, Thomas A. Deméré, Agnese Lanzetti, Annalisa Berta, Mark S. Springer, Robert W. Boessenecker, John Gatesy

**Affiliations:** 1 Department of Biology, San Diego State University, 5500 Campanile Drive, San Diego, CA 92182 USA; 2 Department of Paleontology, San Diego Natural History Museum, 1788 El Prado, San Diego, CA 92101 USA; 3 Paleontology Department, Bargas Environmental Consulting, 3111 Camino del Rio N, Suite 400, San Diego, CA 92108 USA; 4 Department of Vertebrate Zoology, Smithsonian National Museum of Natural History, MRC 108, PO Box 37012, Washington, DC 20013-7012 USA; 5 Imaging and Analysis Center, The Natural History Museum, London, SW7 5BD UK; 6 Department of Evolution, Ecology, and Organismal Biology, University of California-Riverside, Riverside, CA 92521 USA; 7 University of California Museum of Paleontology, University of California, Berkeley, CA 94720 USA; 8 Division of Vertebrate Zoology, American Museum of Natural History, Central Park West at 79th Street, New York, NY 10024 USA; 9 School of Geography, Earth and Environmental Science, University of Birmingham, Birmingham, UK

**Keywords:** Palaeontology, Zoology

**arising from**: C. M. Peredo et al.; *Scientific Reports* 10.1038/s41598-022-15684-8 (2022).

## Introduction

A recent paper by Peredo et al.^1^ criticized the well-established hypothesis that lateral palatal foramina (LPF) on the hard palate of baleen whales (Artiodactyla, Cetacea, Mysticeti) indicate the presence of baleen in extinct species, citing examples of foramina in non-mysticete cetaceans and terrestrial artiodactyls. Peredo et al. argued that all 73 artiodactyl species that they examined have LPF, that terrestrial artiodactyls have just as many LPF as baleen whales, and concluded that these foramina are problematic for predicting the presence of baleen in transitional stem mysticetes^1^. However, we contend that Peredo et al.’s inference is not supported due to misidentification of key anatomical structures, conflation of anatomically divergent palatal foramina in distantly related taxa, artificial similarity between the anatomies of an extinct mysticete and an extant hippopotamus, and gross miscounts of palatal foramina in different species.

The LPF of baleen whales transmit branches of the superior alveolar vessels/nerves, which nourish/innervate baleen in extant mysticetes^2^. The same neurovascular structures nourish/innervate maxillary teeth in odontocetes, but without LPF^3^. LPF that connect internally to the infraorbital canal (IOC) and superior alveolar canal (SAC) also were described for *Aetiocetus weltoni*, a well-preserved Oligocene toothed mysticete that is closely related to crown Mysticeti^4–7^, wherein the SAC connects to both LPF and teeth^3–5^. Specifically, *A. weltoni* has 15 documented LPF (most with sulci)^3^ that are medial to the maxillary toothrow and, as in extant mysticetes^2,4^, are generally symmetrically arranged (8 on left maxilla and 7 on right maxilla; see Supplemental Materials).

**Figure 1:**
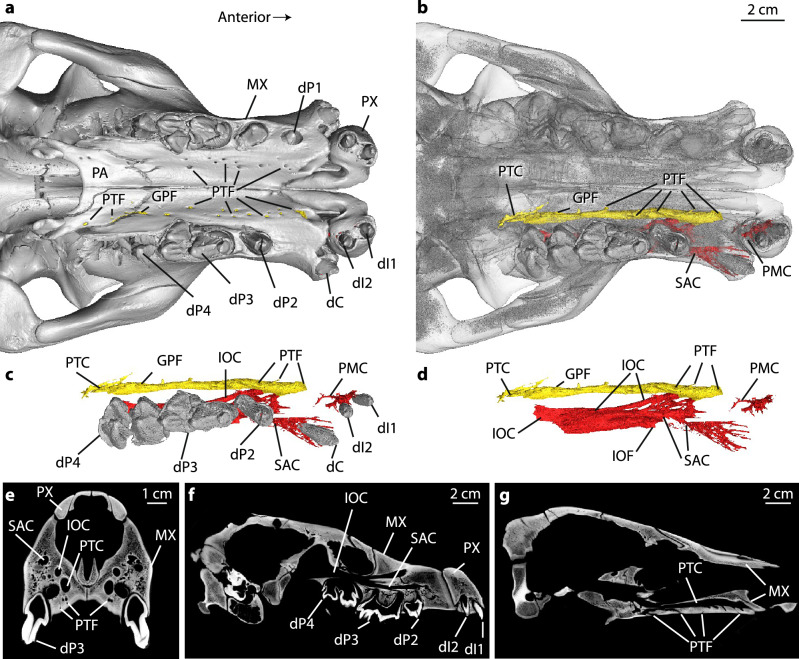
Digital rendering of skull and segmentation of rostral canals of *Hippopotamus amphibius* (UMMZ 101782). (**a**) Skull in ventral view showing palatal foramina. (**b**) Skull rendered semi-transparent to reveal digital segmentation of internal canals. (**c**) Segmentations of left rostral canals and teeth in ventral view with cranium removed. (**d**) Segmentations of left rostral canals in ventral view with teeth and cranium removed. (**e**) Oblique CT slice through rostrum at level equivalent to slice in Fig. 3b of^1^; (**f**) Parasagittal CT slice along tooth row and infraorbital canal. (**g**) Parasagittal CT slice along palatine canal. Anterior is to the right in panels (**a–d,f–g**), dorsal is in up direction in panel (**e–g**). *dC* deciduous canine, *dI1 *deciduous first incisor, *dI2 *deciduous second incisor, *dP1 *deciduous first premolar, *dP2 *deciduous second premolar, *dP3 *deciduous third premolar, *dP4 *deciduous fourth premolar, *GPF *greater palatine foramen, *IOC *infraorbital canal, *IOF *infraorbital foramen, *MX *maxilla, *PA *palatine, *PMC* premaxillary canal, *PTC *palatine canal (identified as SAC in^1^), *PTF *palatine foramina (identified as LPF in^1^), *PX *premaxilla, *SAC *superior alveolar canal. Institutional abbreviations in Supplemental Materials.

**Figure 2:**
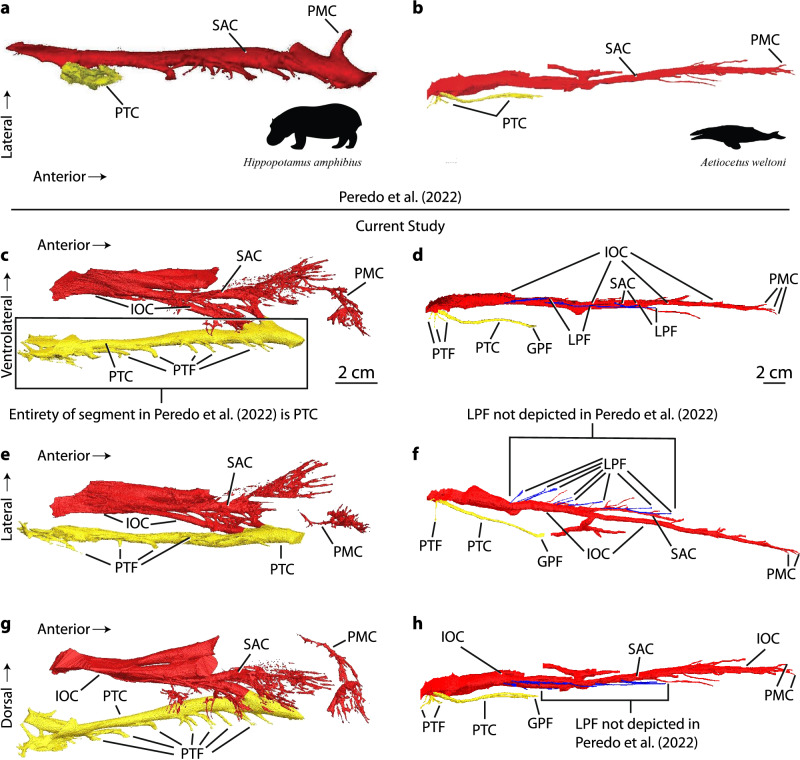
Comparison of the rostral canals of *Hippopotamus amphibius* (UMMZ 101782) and *Aetiocetus weltoni* (UCMP 122900). Digital segmentations of rostral canals of (**a**) *H. amphibius* and (**b**) *A. weltoni* as presented in Fig. 5c, d of^1^. *A. weltoni* modified by Peredo et al. from Fig. 2B of^3^. Digital segmentations of *H. amphibius* (**c,e,g**) from the current study and *A. weltoni* (**d,f,h**); (**c–d**) ventrolateral, (**e–f**) ventral, and (**g–h**) lateral views. Images of left canal segmentations in **c–h** reflected to ease comparison with images of right canal segmentations from^1^. Peredo et al.’s entire segmentation of *H. amphibius* (**a**) is included in the structure rendered yellow in segmentations of the current study (**c,e,g**), indicating that critical neurovascular canals through the rostrum, such as the IOC that connects to the infraorbital foramen and canals that have connections to dental alveoli (e.g., the SAC), were not imaged. There are no direct connections between what Peredo et al. identified as the SAC and the cheek teeth in their segmentation of *H. amphibius*. Comparisons of *A. weltoni* in panels **b, d** and **h** reveal that Peredo et al. juxtaposed canals of *H. amphibius* in ventrolateral view with canals of *A. weltoni* in lateral view. Comparison of *A. weltoni* in (**b,h)** reveals that LPF and associated canals rendered in blue in Fig. 2B of^3^ are not depicted in Fig. 5d of^1^; see also Fig. S3. *GPF *greater palatine foramen, *IOC *infraorbital canal, *LPF *lateral palatal foramina and associated canals (rendered in blue; not depicted in^1^), *PMC *premaxillary canal (labeled as rostral canals in^1,3^), *PTC *palatine canal (identified as SAC in *H. amphibius* in^1^), *PTF *palatine foramina, *SAC *superior alveolar canal. Images from^1^ licensed under a Creative Commons Attribution 4.0 International License (http://creativecommons.org/licenses/by/4.0/). Labels and directional arrows on images of^1^ (**a,b**) reformatted to agree with figures of the current study, but identifications of structures are not changed.

**Figure 3:**
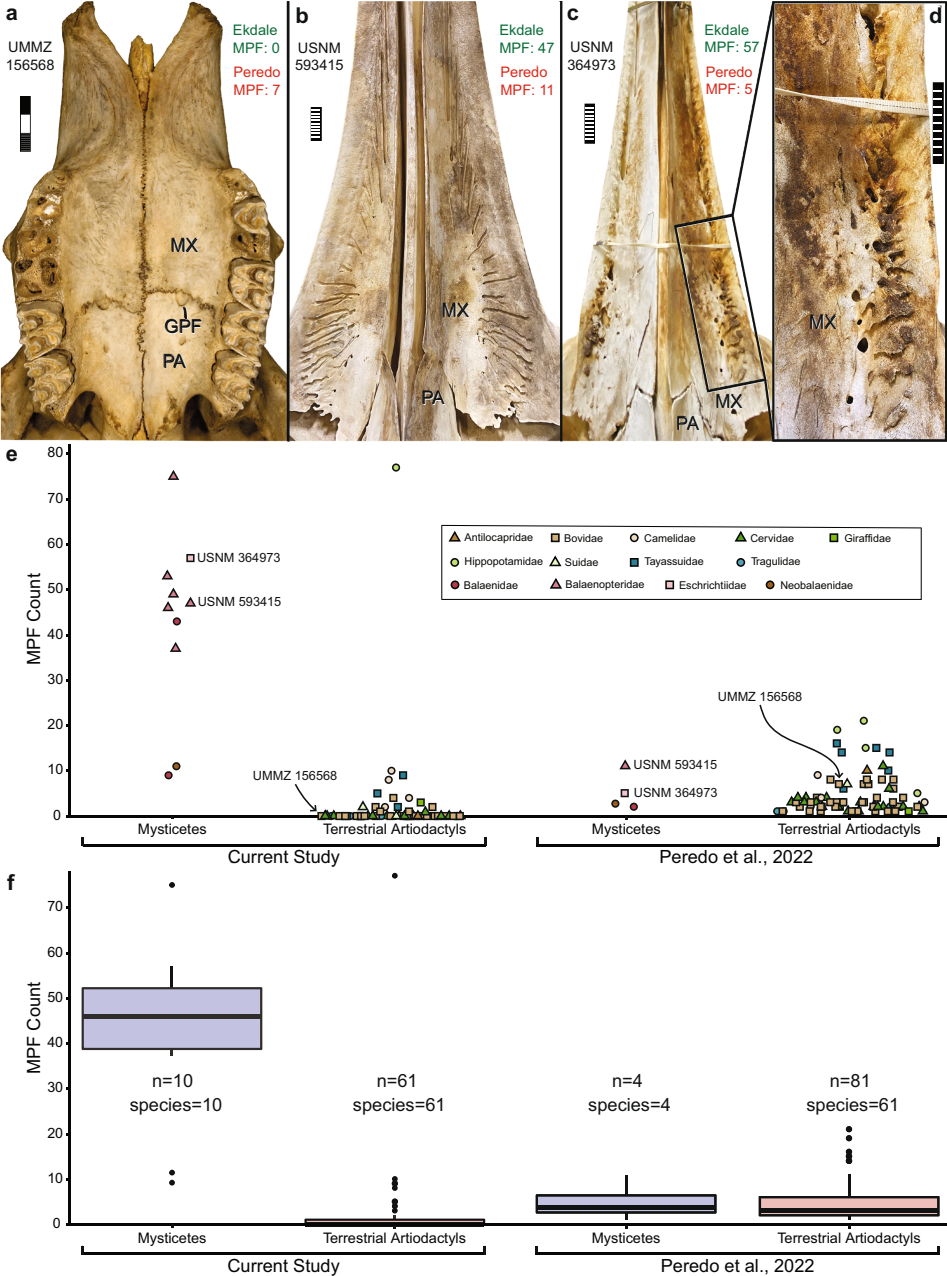
Comparison of the palates of water buffalo (*Bubalus bubalis,* UMMZ 156568) (**a**), sei whale (*Balaenoptera borealis,* USNM 593415) (**b**), and gray whale (*Eschrichtius robustus*, USNM 364973) (**c**,**d**) specimens used in^1^. Note the numerous major palatal foramina ≥ 1 mm (MPF) and associated sulci on the maxillae of the two mysticetes (**b**–**d**) and absence of MPF, as well as sulci, on the maxilla of the water buffalo (**a**), except for the two greater palatine foramina symmetrically positioned at the maxillopalatine suture. Disparate counts of MPF from^1^ (red) and from the current reanalysis for the maxilla (green) are shown for the three specimens. (**e**) Plot of MPF counts for each mysticete and terrestrial artiodactyl specimen from our reanalysis compared to Peredo et al. Specimens figured here (**a–d**) are identified by specimen numbers. Colors and shapes correspond to family-level association of each specimen. Horizontal spread of data within each bin is only to reduce overlap of data points. (**f**) Boxplot summaries of MPF-count data from the current study and from^1^. Numbers of specimens and species used in each study are shown. Note the significantly higher median value for mysticetes and lower median value for terrestrial artiodactyls in the current reanalysis relative to Peredo et al., completely contradicting one of Peredo et al.’s main conclusions that “mysticetes do not exhibit an increased number of palatal foramina…relative to that of terrestrial artiodactyls” (p. 7 of^1^). For the current analysis, MPF-counts in mysticetes and terrestrial artiodactyls are significantly different (Tukey’s Honest Significant Difference test: p = 3.3e^−15^, F = 101.6, df = 1). *GPF *greater palatine foramen, *MX *maxilla, *PA *palatine. Scale bar for (**a**) is 3 cm with mm increments on first cm, and scale bars for (**b–d**) are 10 cm. Photo of *Bubalus bubalis* provided by P. Myers.

Using CT scans, Peredo et al. reported internal connections between palatal foramina and the SAC in several terrestrial artiodactyls, including *Hippopotamus amphibius*, and argued that these foramina are homologous to LPF of mysticetes. To test this assertion, we segmented major rostral canals in *H. amphibius* using the same CT dataset as^1^. Peredo et al. did not segment critical canals in lateral regions of the rostrum that contradict their assertion that LPF are present in *H. amphibius* (Figs. 1 and 2, S1, Video S1). The canal identified as the “SAC” (Figs. 3, 5 in^1^) does not include branches connecting directly to the teeth/alveoli as expected for a canal carrying alveolar neurovasculature, so it is not clear to us why Peredo et al. identified this canal as the SAC. Instead, the canal identified by Peredo et al., which connects to the palatal foramina, is more likely the canal for palatine neurovasculature given its direct connection to the greater palatine foramen (Fig. 1a–g). We identified the true SAC in *H. amphibius* as a distinct, more lateral canal with clear connections to the IOC and the maxillary alveoli but with no connections to the prominent palatal foramina (Fig. 1b–g; Fig. S1). When we reconstructed the internal connections of palatal foramina using Peredo et al.’s CT datasets for three additional terrestrial artiodactyls and an unpublished dataset for *Tayassu pecari* (Fig. S2), we again found that Peredo et al. misidentified the SAC in *Sus scrofa* and *Lama glama* and that all prominent palatal foramina connect to palatine canals in all species that we examined (Supplemental Materials). Discordant internal ‘plumbing’ of palatal foramina across Artiodactyla demonstrates that the palatal foramina in terrestrial species are not anatomical homologs to the LPF that connect to the SAC in living and extinct mysticetes.

Nonetheless, Peredo et al. argued that “3D models show that the morphology observed in stem mysticetes…is consistent in terrestrial artiodactyls” (p. 5 of^1^) by juxtaposing their segmentations of the misidentified rostral canals of *Hippopotamus amphibius* with a modified image of rostral canals in *Aetiocetus weltoni* (Fig. 5c, d of^1^; Fig. 2a, b). Specifically, lateral palatal canals that lead to LPF in a published image of *A. weltoni* (Fig. 2B of^3^) are not depicted in Fig. 5d of Peredo et al. (Fig. 2, Fig. S3), despite these structures appearing in the original image and being the primary subject of both papers. Missing segmentations of the more lateral rostral canals (SAC, IOC) in *H. amphibius*, absence of LPF canals in the *A. weltoni* image, and comparison of specimens in incompatible views yielded spurious morphological consistency, which was paramount to Peredo et al.’s conclusions (Fig. 2). Additional inaccuracies regarding cetacean data and paleontological literature are provided in the Supplemental Materials.

In aetiocetid mysticetes, LPF are expressed externally as ~ 1 mm foramina in the maxilla medial to cheek teeth and alveoli^3–5^. However, in their examination of 61 terrestrial artiodactyl species, Peredo et al. identified some foramina as LPF “in the inter-alveolar septae, the alveolar margins, and even within the alveoli of missing teeth” (p. 3 of^1^). They also identified foramina in the premaxilla of two “archaeocete” cetaceans as LPF (Fig. 4 of^1^, Fig. S3A of^8^), again revealing that they conflated maxillary LPF in mysticetes with non-homologous neurovascular structures in other taxa. In both crown and stem mysticetes, LPF typically are associated with elongate sulci^2–5^. However, such sulci (Fig. 3b–d) were not described in detail by Peredo et al. for terrestrial artiodactyls, even though they represent a key feature of LPF anatomy in mysticetes and are absent from the palatal foramina of most terrestrial artiodactyl species that we examined (Figs. S4–S6; Table S2).

Central to Peredo et al.’s argument is that “mysticetes do not exhibit an increased number of palatal foramina…relative to that of terrestrial artiodactyls” (p. 7 of^1^), so LPF cannot be used to infer baleen in early stem mysticetes. However, they quantified “total number of clusters of palatal foramina” (TPF) and “major palatal foramina” (MPF) without providing a methodology for doing so. It is unclear whether their foramina counts were limited to those on the maxillae medial to the tooth row or to all foramina on the palate (see above). We counted MPF (≥ 1 mm as in^1^) on the maxilla across 100 artiodactyl species (including 4 mysticete and 4 terrestrial artiodactyl specimens analyzed in^1^, as well as 62 of 66 extant species and all 52 extant genera in^1^) using our methodology as described in the Supplemental Materials. Our counts were drastically different from Peredo et al. (Table S2; Table S1 of^1^). For example, utilizing the same specimens as in^1^, we counted 57 MPF in *Eschrichtius robustus* (USNM 364973) whereas Peredo et al. reported just 5; for *Balaenoptera borealis* (USNM 593415) we counted 47 MPF whereas Peredo et al. reported only 11; and for *Bubalus bubalis* (UMMZ 156568) we counted 0 MPF whereas Peredo et al. reported 7 (Fig. 3). Given that mysticete LPF are so large and easily observed, Peredo et al.’s MPF counts are inexplicably low (Fig. 3b–d, Fig. S6), but for terrestrial artiodactyls (e.g., Fig. 3a), their counts are systematically high relative to ours (Fig. 3e, f). As further examples, in the same specimens of *Aepyceros melampus* (UMMZ 124571), *Antilocapra americana* (UMMZ 65502), and *Damaliscus pygargus* (UMMZ 167702), we counted 0 MPF on the maxillae for each, while Peredo et al. reported 8, 10, and 6, respectively. In fact, 45 of 61 terrestrial artiodactyl species that we examined lacked MPF on the maxilla, and 57 of 61 species had ≤ 5 MPF, whereas all mysticetes had numerous LPF (range 9–75; median 46.5) as noted previously^4,5^. By contrast, Peredo et al. recorded MPF in all 61 terrestrial artiodactyls they examined and found that MPF-counts in mysticetes and terrestrial artiodactyls are not significantly different^1^. However, our reanalysis revealed a highly significant difference (p = 3.3e^−15^) using the same statistical test as in^1^ due to the numerous, large LPF on mysticete palates (Fig. 3b–f, Fig. S6).

In conclusion, we contend that terrestrial artiodactyls do not have LPF as in mysticetes. Re-examination of CT datasets of^1^ reveals that Peredo et al.’s prominent “LPF” in terrestrial artiodactyls connect to the same internal canal as the greater palatine foramen and not to the IOC or SAC as in mysticetes (Figs. 1, 2, Figs. S1, S2). There are no direct connections between these palatine canals and the cheek teeth or dental alveoli. Moreover, the great majority of terrestrial artiodactyls have no MPF on the maxilla (Fig. 3a, e, Figs. S4, S5; Table S2) and commonly lack sulci associated with MPF (Fig. 3a, Figs. S4, S5; Table S2). Furthermore, even in cases where maxillary MPF are present and show superficial resemblance to the generally symmetrical array of LPF in mysticetes (e.g., *Hippopotamus amphibius*, *Lama glama*, *Tayassu pecari*), homology with mysticete LPF is not supported (Figs. 1, 2, Figs. S1, S2). Given our reanalysis, it remains the simplest explanation of the current evidence to interpret the phylogenetically, positionally, and structurally homologous LPF of mysticetes as indicators of baleen in stem mysticetes^3–5,9^.

### Supplementary Information

Supplementary Information.

Supplementary Table S2.

Supplementary Video 1.

## Data Availability

Links to CT data used both here and by Peredo et al. are available in^1^. Information regarding CT data of *Tayassu pecari* are provided in Supplemental Text S1.
