# Supplementary material for: Lateral palatal foramina are not widespread in Artiodactyla and imply baleen in extinct mysticetes
Source: Sci Rep. 2024 May 3;14:10174. doi: 10.1038/s41598-024-60673-8 (PMC11068900; doi:10.1038/s41598-024-60673-8)
Supplement: Supplementary file 1 — Supplementary Information. [file 41598_2024_60673_MOESM1_ESM.pdf]

Supplemental Materials for:

Lateral palatal foramina are not widespread in Artiodactyla and imply baleen in extinct mysticetes

(Ekdale, El Adli, McGowen, Deméré, Lanzetti, Berta, Springer, Boessenecker, and Gatesy)

<u>Contained in this file:</u>

Supplemental Text S1. Extended description of rostral and palatal anatomy.
Supplemental Text S2. Methods for counting major palatal foramina (MPF).
Supplemental References. References cited in Supplemental Materials that do not appear in main text of the article.
Figure S1. Additional digital rendering of skull and segmentation of rostral canals of *Hippopotamus amphibius* (UMMZ 101782) in multiple views.
Figure S2. CT slices through rostrum of terrestrial artiodactyls.
Figure S3. Comparison of 3D renderings of rostral canals of *Aetiocetus weltoni* (UCMP 122900).
Figure S4. Left half of the palate of terrestrial artiodactyl specimens used in [1] showing the condition of maxilla and palatine, along with Peredo et al.'s MPF counts (red text).
Figure S5. Palates of *Gazella* spp. showing the condition of premaxilla, maxilla, and palatine.
Figure S6. Palates of cetaceans showing LPF and condition of maxilla.
Table S1. Parameters of CT data of terrestrial artiodactyls.

<u>Contained in separate files:</u>

Table S2. MPF data for terrestrial artiodactyls and cetaceans.
Video S1. Rotating animation of digital rendering of skull of *Hippopotamus amphibius* (UMMZ 101782), skull rendered semi-transparent to visualize rostral canals, skull removed leaving only segmentations of rostral canals with teeth. Red segments represent the IOC and SAC. Yellow segments represent palatine canals with branches to palatal foramina.

Supplemental Text S1. **Extended description of rostral and palatal anatomy.**

We re-examined the CT datasets of *Hippopotamus amphibius* (UMMZ 101782), *Lama glama* (TMM M-2052), *Odocoileus virginianus* (USNM VZ 118627), and *Sus scrofa* (USNM VZ 260907) that were made available by Peredo et al. [1]. In addition, we analyzed an unpublished dataset of *Tayassu pecari* (USNM 406851). This taxonomic sampling includes representatives of five terrestrial artiodactyl clades (Hippopotamidae, Camelidae, Cervidae, Suidae, Tayassuidae) that display divergent palatal anatomies. The CT dataset of the peccary was downloaded from www.MorphoSource.org (media 000100741), Duke University. Digital segmentations of the rostral canals in all datasets were performed in Avizo 3D software [10], and CT slices were resliced using ImageJ 1.53t. Specific scanning parameters, including image resolution and voxel dimensions, that were not reported by Peredo et al. are provided in Table S1. In addition, we discovered that the numbers of slices for *O. virginianus* and *S. scrofa* were lower than reported by Peredo et al., and those errors have been fixed here. Institutional abbreviations used in Supplemental Text S1: **TMM M** – Texas Natural Science Center, Austin, TX; **UCMP** – University of California Museum of Paleontology, Berkeley, CA; **UMMZ** – University of Michigan Museum of Zoology, Ann Arbor, MI; **USNM**– National Museum of Natural History, Smithsonian Institution, Washington, DC.

Rostral Canals of *Hippopotamus amphibius* (UMMZ 101782) - Hippopotamidae (Figure S1, Video S1)

The specimen of river hippo is a juvenile, but the morphology described below serves as a baseline for future anatomical investigations. There are two small incisors in each premaxilla, and the canine and three to four premolars within each maxilla; the dP1 is missing on the left side, which is either a pathology or the deciduous tooth has already been shed. The premolars are not fully erupted and the dP4 on each side is still in the crypt.

In ventral view (Figure 1 in main text), numerous circular to oval foramina penetrate the palatal processes of the palatine and maxilla. Four foramina are observed penetrating the left palatine, and five are observed through the right palatine. The palatal foramina in each maxilla are arranged in two parasagittal rows, medial to the tooth row, and extending from the maxillopalatine suture to the anteromedial section of the maxilla. Examination of the CT dataset referenced by Peredo et al. confirms that each of the palatal foramina, including those penetrating both the maxilla and palatine, connect internally to a common bony canal that Peredo et al. identified as the superior alveolar canal (SAC).

We digitally segmented the major bony canals and their secondary branches that extend the length of the left side of the rostrum (Figure S1; Video S1). It is important to note that we reconstructed the courses of bony canals through the rostrum, not the soft tissue neurovasculature that the canals transmit. Dissections and/or vascular injections will be needed to make ultimate identifications, as has been done for other terrestrial artiodactyls [11–13].

Our general strategy for identifying the segmented canals in the CT scan for the hippo was straightforward. We began by tracing canals that extended into the rostrum from the infraorbital foramen, from the teeth/alveoli, from the greater palatine foramen, and from other prominent palatal foramina. Such easily identified landmarks provide a solid anatomical framework for all downstream interpretations of homology: 1) the infraorbital canal (IOC) exits the rostrum at the infraorbital foramen; the IOC is a critical canal from which the SAC branches in many mammals; 2) tracing rostral canals that connect to the teeth/alveoli, by definition, should lead to the IOC and/or SAC that transmit neurovasculature to the dentition in mammals; 3) the greater palatine foramen connects to palatine canals in the rostrum of mammals, so tracing the greater palatine foramen into the rostrum should distinguish palatine canals from the IOC and SAC; 4) segmentation of these rostral canals then provides anatomical context for tracing the internal interconnections of all remaining palatal foramina and determining whether these connect to the SAC and IOC as in lateral palatal foramina (LPF) of mysticetes. Any additional major rostral canals and their interconnections/pathways were also segmented and interpreted following this solid foundation.

Through our reanalysis of the CT data, we identified three subparallel canals extending the length of the rostrum: medialmost is the canal for greater and lesser palatine vessels and nerves (abbreviated as PTC), lateralmost is the SAC, and in between is the IOC. Our re-examination of the CT data reveals that Peredo et al. misidentified the PTC as the SAC and in so doing misidentified medially positioned palatine foramina (PTF) that connect to the PTC as LPF, like those that connect to the IOC and SAC in mysticetes. The PTC does not connect directly to cheek teeth, so it is not clear why Peredo et al. identified this canal as the SAC.

Canal for greater and lesser palatine vessels and nerves (PTC): The posterior opening of the PTC is within the pterygopalatine fissure, which is medial to the dP4 crypt (Figures 1, S1, Video S1). There is no bony connection of the PTC to the maxillary canal or to the IOC posterior to its entrance into the rostrum. Immediately anterior to the entry of the PTC into the palatine, multiple canals descend ventrally to open as the foramina observed on the palatal surfaces of the palatine and maxilla. In general, most of the PTF connect to the PTC via anteroventrally directed canals (Figure S1d–f), and many appear to be paired. In other words, two canals depart the PTC close to one another (Video S1). This pattern is mirrored on both right and left sides of

the palate. The PTC as a whole enlarges in diameter as it passes medial to the dP3. The anterior terminus of the PTC opens as a very large anterior palatine foramen at the anteromedial section of the maxilla with a broad sulcus leading to the incisive foramen within the maxillopremaxillary suture (Figure 1a,b). There are no direct connections between the PTC and maxillary alveoli. However, a more laterally positioned canal that we identify as the IOC (see below) joins with the PTC medial to the root of dP2 (Figure S1b,e). Anterior to this junction is a narrow canal that connects with the even more laterally positioned SAC.

Infraorbital canal (IOC): The posterior opening of the IOC is situated within the orbital fossa and extends anteriorly dorsal to the dP4 crypt (Figures 1, S1, Video S1). At a transverse level between dP3 and dP4, the IOC divides into four passages (Figure S1b,e,h): two lateral branches (leading to infraorbital foramina), one medial branch (continuation of IOC), and one intermediate branch (SAC). The ventralmost of the two lateral canals that open as infraorbital foramina on the lateral surface of the maxilla sends numerous fine canals that contribute to the porous nature of the spongy bone surrounding the posterior premolar alveoli. The medial branch (IOC) extends anteriorly before ultimately joining with the PTC medial to the anterior root of the dP2 (see above; Figure S1b,e, Video S1).

Superior alveolar canal (SAC): The intermediate branch (SAC) departing from the IOC extends dorsal to the premolars and sends numerous fine canals to the premolar alveoli (Figure S1e,h). We identify the canal as the SAC given that it connects to maxillary alveoli and teeth via small dental or individual alveolar canals (Figure S1, Video S1) as observed in the toothed mysticete *Aetiocetus weltoni* and extant odontocete *Tursiops truncatus* [3]. The anterior terminus of the SAC is composed of a network of fine canals that open within and around the canine alveolus. There are several cross-connections between the SAC, IOC, and PTC, particularly a branch anteromedial to the dP2 that connects the SAC to the PTC (Figure S1b,h, Video S1).

Premaxillary canals (PMC): There are no bony connections between any of the maxillary canals and the PMC (Figures 1, S1). A small foramen that opens on the external surface of the premaxilla at a horizontal level with the more dorsal of the two infraorbital foramina (Figure S1g) leads to a network of PMC within the bone. A narrow canal extends anteriorly from the foramen for a short distance, and then curves ventrally before bifurcating and ultimately proliferating into the spongy bone that surrounds the incisor alveoli (Figure S1e,h, Video S1). Although there are no bony connections between maxillary and premaxillary canals of the hippopotamus, dissections of the arteries and veins of the head might reveal a soft tissue connection between the maxillary and premaxillary vasculature. In some terrestrial artiodactyls, a delicate branch of the infraorbital artery exits the infraorbital foramen and enters the premaxilla via a small canal similar to that in the hippo [11–13]. The small artery in these

terrestrial species ultimately nourishes the incisors, and it is likely that the arterial blood follows a similar course to the incisors in the hippo.

Rostral Canals of *Lama glama* (TMM M-2052) - Camelidae (Figure S2a–c)

As with the hippo, several foramina penetrate the palatal processes of the palatine and maxilla in the llama. Analysis of the CT slices of the llama reveal two major canals extending in an anterior-posterior direction through the rostrum (Figure S2a). The palatal foramina in both palatines and maxillae, including the greater palatine foramen, connect internally to the more medial of the two canals (Figure S2c). We identify the medial canal as the PTC, which carries the greater and lesser palatine vessels in the closely related alpaca, *Lama pacos,* wherein injection of high contrast media and CT scanning have revealed the courses of the maxillary artery and its branches through the rostrum [12]. In our reconstructions, there are no direct connections between the PTC and the maxillary alveoli, as is expected for the PTC. The more lateral of the two canals is the IOC and it is situated immediately dorsal to the maxillary dentition (Figure S2b). The IOC opens anteriorly through the infraorbital foramen. There is no SAC in the llama. Rather, the maxillary teeth are nourished by branches of the infraorbital artery within the IOC [12]. In fact, our reanalysis of the llama dataset revealed that the structure labeled as the SAC in fig. 2 of [1] is instead a pneumatic space within the spongy bone surrounding M1 (Figure S2a).

Rostral Canals of *Odocoileus virginianus* (USNM VZ 118627) - Cervidae (Figure S2d–f)

There are several small foramina through the palate that connect internally to the maxillary sinuses and spaces within spongy bone surrounding the cheek teeth, although they are not as pronounced as in the other terrestrial species that Peredo et al. CT scanned. The small openings occur at the base of the teeth and are similar to miniscule foramina that we observed in other terrestrial artiodactyl species (Supplemental Text S2). The CT slices reveal two major bony rostral canals that are surrounded by an extensive maxillary sinus (Figure S2d). The more medial of the two canals is the PTC that connects internally with the greater and lesser palatine foramina (Figure S2f). As in the other terrestrial artiodactyls that we examined, there are no direct connections between the PTC and the maxillary teeth. The lateral of the two canals, which is distinctly oval in cross-section, is the IOC, which opens anteriorly as the infraorbital foramen. The IOC is situated dorsal to the medial roots of the molars (Figure S2d,e). There is no SAC in the deer, and it is possible that: 1) in the absence of a superior alveolar artery, the maxillary teeth are nourished directly by branches of the infraorbital artery within the IOC and/or maxillary sinuses, or 2) the superior alveolar artery travels alongside the infraorbital artery within the same bony canal (IOC) and connects to the teeth via small branches. In either case, examination of the soft tissue vasculature is needed to make such a determination.

Rostral Canals of *Sus scrofa* (USNM VZ 260907) - Suidae (Figure S2g–i)

The domestic pig has several distinct foramina penetrating the palate, including the opening labeled as a LPF in fig. 2 of [1]. Owing to the fused nature of the palatal elements, it is difficult to identify the bone that each foramen penetrates. The CT slices reveal that not only does the structure labeled as LPF by Peredo et al. connect internally to the PTC, but it is also posterior to (and in line with) the greater palatine foramen (Figure S2i). This contrasts with their assertion that “LPF” in non-mysticetes “are positioned well anterior to the greater palatine foramen” (p. 7 of [1]). The PTC is the medial of two major canals extending through the rostrum of the pig (Figure S2g), and it has no direct connection to the maxillary dentition. The second of two major rostral canals is the IOC and it is situated immediately dorsal to the maxillary dentition before opening anteriorly as the infraorbital foramen (Figure S2g–h). In our reconstruction, there is no SAC in the pig. Instead, the maxillary teeth are nourished by “dental branches” of the infraorbital artery within the IOC according to previous dissections of soft anatomy (p. 1314 of [11]).

Rostral Canals of *Tayassu pecari* (USNM 406851) - Tayassuidae (Figure S2j–l)

Peredo et al. did not CT scan a peccary, although they reported large numbers of MPF in different peccary species relative to most terrestrial artiodactyls [1]. We examined a CT dataset for the white-lipped peccary, and indeed the palate is penetrated by numerous large foramina. It is difficult to determine which bone the foramina penetrate owing to extensive palatal fusion in peccaries, but all of the palatal foramina medial to the toothrow connect internally to the same bony canal that we identify as the PTC (Figure S2l). The PTC, which connects directly to the greater palatine foramen, is the medial of three major rostral canals, and it is medial to the maxillary dentition (Figure S2j). There are no direct bony connections between the PTC and the teeth, so we do not consider this canal to be the SAC. The large IOC extends dorsal to the lateral roots of the cheek teeth (Figure S2j–k). Numerous canals depart the ventral surface of the IOC and open into the molar alveoli. The IOC opens onto the lateral surface of the skull via the infraorbital foramen at a level posterior to the P3. The ventral border of the infraorbital foramen continues anteriorly as a sulcus with two foramina. The posterior foramen leads to a medially directed canal that we identify as an extension of the IOC (Figure S2j–k) that ultimately connects with the PTC medial to P1. The anterior foramen within the sulcus of the infraorbital foramen leads to an anteriorly directed canal that curves dorsally and opens into a large sinus posterior to the canine root. Multiple canals lead from the sinus to a spongy region surrounding the canine, as well as to the canine alveolus itself. We tentatively identify this canal as the SAC (Figure S2j).

Palatal foramina in Odontoceti, ‘Archaeoceti’, and stem mysticetes

Peredo et al. cited several examples of purported LPF in odontocetes, ‘archaeocetes’, and stem mysticetes. These occurrences have been discussed elsewhere [3–9] and challenged/refuted by us in two of these papers [3,9], but further clarification is warranted.

For Odontoceti, Peredo et al. sampled just one extant (*Tursiops truncatus*) and three extinct species (*Isthminia panamensis*, *Xenorophus sloanii*, *Simocetus rayi*). Ekdale and Deméré [3] noted that the SAC of *T. truncatus* “diverges from the infraorbital canal anterior to the branching of the palatine canal and transmits superior alveolar vessels and nerves to the maxillary alveoli and teeth via dental/alveolar canals” (p. 400 of [3]). It is clear from the CT slice through the rostrum of *T. truncatus* in fig. 2 of [1] that the anatomical structure identified by Peredo et al. as a canal connecting to a “LPF” is within the spongy alveolar bone, like the dental canals leading to teeth and alveoli in the *T. truncatus* specimen examined by Ekdale and Deméré (see fig. 5a of [3]). A similar pattern in which dental canals connect the SAC to alveoli was described for *Aetiocetus weltoni.* However, the SAC of *A. weltoni* additionally connects to LPF via lateral palatal canals, which are absent in *T. truncatus* [3]. Peredo et al. counted palatal foramina in the extinct crown odontocete *Isthminia panamensis* (Inioidea), recorded two MPF, but neither figured nor made any reference to CT scans of this specimen (USNM PAL 546125).

Regarding stem odontocetes, the canal that Peredo et al. identified as connecting to LPF in the stem odontocete *Xenorophus sloanii* in fig. 2 of [1] is associated with alveolar and dental vasculature, rather than a palatal structure medial to the teeth. Peredo et al. also reported “LPF” with associated sulci in a second stem odontocete, *Simocetus rayi* (fig. 4 of [1]). As we demonstrate in our current study, palatal foramina that are superficially similar to LPF in mysticetes do not necessarily connect to the IOC or SAC as revealed by CT data. Peredo et al. did not provide CT evidence of such connections in *Simocetus*. In fact, the openings were originally described as anterior palatine foramina by [14] (see discussion by [3]), and we tentatively accept that identification until more compelling data suggest otherwise. We also note that the anteriormost “LPF” and “sulcus” identified in fig. 4b of [1] for *Simocetus rayi* is a fracture in the specimen and not part of the animal’s anatomy.

The single extant odontocete sampled by Peredo et al. precludes any meaningful quantitative analyses at this time, but in this brief reply to [1], we did characterize palatal foramina and associated sulci in 29 extant odontocete species across 25 genera (Supplemental Text S2). This much larger taxonomic sampling compared to [1] records a broad diversity of palatal anatomies within crown Odontoceti (Table S2). Our ongoing work on this clade (Ekdale et al., in prep.),

including detailed segmentations of CT scans with sampling at the family level, is necessary to determine the homology of medial and more laterally positioned palatal foramina and sulci in different species (Table S2). As we have demonstrated for terrestrial artiodactyls (Figures 1–2, S1–S2), accurately tracking the “internal plumbing” of palatal foramina is critical for sorting out the evolutionary history of these structures. Currently, convincing anatomical evidence for the presence of LPF in odontocetes is lacking.

Peredo et al. examined three ‘archaeocete’ (stem) cetaceans in their study. The partial rostrum of *Basilotritus wardii* that was studied by Peredo et al. (USNM PAL 310633) only “includes left and right premaxillae and part of the right maxilla” (p. 514 of [15]). Our reanalysis of the *B. wardii* CT dataset revealed that the canal labeled in fig. 2 of [1] is within the premaxilla. As has been discussed extensively [2–5,9], LPF have been found in the maxilla only, and recognition of palatal foramina on the premaxilla of *B. wardii* is a conflation of neurovascular structures that are not homologous with LPF in mysticetes. For *Dorudon atrox* and *Zygorhiza kochii*, palatal foramina were identified previously as anterior palatine foramina (see discussions in [3]). We also note that the structures identified for *D. atrox* in fig. 4b of [1] are primarily in line with the toothrow in the interdental septum and not medial to the toothrow; this contrasts with the condition in aetiocetid mysticetes. Furthermore, the anteriormost foramen and sulcus identified in fig. 4b of [1] is clearly within the premaxilla. As discussed above for *B. wardii*, LPF have only been found in the maxilla of mysticetes [2–5,9]. Overall, the evidence for presence of LPF in ‘archaeocete’ cetaceans is currently uncompelling.

Peredo et al. also argued for the presence of LPF in five stem mysticetes (including *Aetiocetus weltoni* and *Aetiocetus cotylalveus*), which they contended extends the distribution of LPF to the deepest lineages of Mysticeti and to some taxa that might have lacked baleen. In so doing, they conflated phenotypically-divergent palatal foramina across a broad taxonomic range by misrepresenting previous anatomical descriptions (see our thorough discussions in [3–5,9]). For example, they incorrectly argued that LPF have been described for the toothed mysticete *Llanocetus denticrenatus* [6]. Although fine “striations” surround some of the upper premolar alveoli of *L. denticrenatus*, “there are no unequivocal foramina” in the palate of the only known specimen (p. 11 in supplemental description in [6]), which is in stark contrast to any LPF that have been described in the well-characterized toothed mysticete *Aetiocetus weltoni* [3–5,9]. Furthermore, Peredo et al. previously coded LPF as present in the edentulous mysticete *Maiabalaena nesbittae* (see data matrix of [8]), but “palatal foramina of *Maiabalaena* do not visibly communicate with the SAC” (p. 3994 of [8]), penetrate ~5mm into the maxilla, and then appear to terminate. This suggests that the dead-end holes are not LPF, are modified LPF in which connections to the SAC have been lost, or that the lateral palatal canals which connect to the SAC are not visible due to inadequate CT resolution [8] (also see discussion in [3,9]).

Coronodon was initially noted to have “only three to four minute, palatal foramina, most of which are clustered around the P3…” (p. 4 of [7]). However, more detailed study of the holotype skull of *C. havensteini* (CCNHM 108) in [16] described nine lateral palatal foramina of >1 mm in diameter, and many have elongate sulci. Though portions of the interior of the holotype skull are damaged, examination of CT data in [16] found that these foramina “traced internally trending posterodorsally to dorsolaterally and towards the roots of the teeth rather than medially” (p. 32 of [16]), supporting their identification as LPF. Presence or absence of LPF in other specimens and species of *Coronodon* have yet to be discussed within the literature.

According to Peredo et al. [1], only a single foramen is present in the palate of the stem mysticete *Aetiocetus cotylalveus*, the type species of *Aetiocetus* (also see discussion in [4–5]). However, our examination of the holotype specimen (USNM 25210) revealed multiple additional palatal foramina and sulci in the maxilla medial to the toothrow. Unfortunately, the holotype was likely preserved using beeswax, which, along with extensive fracturing of the palate, obscures fine surficial detail of small neurovascular canals. Regardless, we observed more LPF/sulci in *A. cotylalveus* than reported in [1], and the low number of foramina recorded by Peredo et al. is better explained by limited preservation of the specimen rather than biological reality. Additional details regarding the number and anatomy of LPF will be revealed through further preparation of the specimen and more thorough analysis of the CT dataset for this specimen (Ekdale et al., in prep.).

More generally, it is unreasonable to think that accurate counts of palatal foramina can be made from fossils that are not exceptionally well preserved and physically prepared, given that foramina ≤1mm in size are challenging to discern when infilled with rock matrix, and are impossible to discern when large sections of the palate are completely missing or not yet prepared. For example, the holotype skull of *Aetiocetus weltoni* is exceptionally well preserved but only eight LPF were initially described because only the left side of the palate has been prepared [8]. Subsequent CT analysis of the specimen revealed another seven LPF on the right side of the palate for a total of 15 [3]. Most early mysticete fossils are not nearly as well preserved (see discussion in [3]), so counts of LPF should be considered minimum estimates, rather than taken at face value as accurate counts, as has been done previously [1,8].

Peredo et al. acknowledged presence of LPF in the Oligocene stem mysticete *Aetiocetus weltoni* [2–5,9], but their misrepresentation of rostral canals in the species presents specious similarity to those in *Hippopotamus amphibius* (fig. 5 of [1] modified from [3]). This false similarity (Figure 2a–b) also is driven by Peredo et al.’s omission of the more laterally positioned IOC and SAC in their CT segmentation of *H. amphibius* (Figure 2). When complete digital renderings of the

rostral canals of *A. weltoni* and *H. amphibius* are presented in the same orientation, we found striking differences between the anatomies of these divergent taxa. Indeed, they look nothing alike (Figure 2c–h). For reference, Peredo et al. presented the canals of *H. amphibius* in ventrolateral view (Figure 2c) and the canals of *A. weltoni* in lateral view (Figure 2h), despite both being shown with the same orientation arrows in [1]. Peredo et al. acknowledged that they modified the image of *A. weltoni* from [3] but provided no details regarding the nature of their modifications. We present a direct comparison of Ekdale and Deméré's [3] original image of rostral canals in *A. weltoni* to Peredo et al.'s modified version in Figure S3. The palatal canals leading to LPF, which are colored blue in the original image (fig. 2b of [3]), are absent in Peredo et al.'s mirrored version (fig. 5d of [1]) of the original two-dimensional figure (Figure S3b). Presentation of the *A. weltoni* image in this fashion is disconcerting, given that LPF and their associated canals were the primary focus of Peredo et al.'s study and omission of these anatomical features resulted in greater support for Peredo et al.'s central hypothesis [1] relative to an alternative hypothesis proposed in our previous work [3,9].

Supplemental Text S2. Methods for counting major palatal foramina (MPF).

The Methods section of [1] does not describe how Peredo et al. measured and counted what they termed “major palatal foramina” (MPF) and “total number of clusters of palatal foramina” (TPF). Their entire Methods section is repeated below, and we note that no methodology was presented for measuring and counting palatal foramina or clusters of foramina in this text (p. 8, 2nd paragraph in [1]):

> “We observed the palates of 16 living and fossil cetaceans and 81 specimens spanning 61 species of extant artiodactyls (Table S1). These specimens are reposited at the University of Michigan Museum of Paleontology (UMMP), the University of Michigan Museum of Zoology (UMMZ), and the Departments of Paleobiology and Vertebrate Zoology at the National Museum of Natural History, Smithsonian Institution (USNM). All specimens were studied with permission for their respective curators and host institutions. We selected a subset of these specimens for CT-scanning and reconstructed the internal morphology associated with the external foramina (Table S2). The CT scanning for *Sus*, *Odocoileus*, *Balaenoptera*, *Basilotritus*, and *Tursiops* was conducted at the Smithsonian Institution Bio-Imaging Research (SIBIR) Center in the Department of Anthropology at the USNM. The CT scanning for *Zygorhiza* and *Xenorophus* was conducted by National Technical Systems located in Belcamp Maryland. The CT scans of *Lama* were conducted at the University of Texas at Austin and were provided courtesy of Digimorph and Timothy Rowe. The CT scans of *Hippopotamus* were conducted at the University of Michigan Museum of Zoology and were provided courtesy of Morphosource and Cody Thompson and Ramon Nagesan. All statistical tests were conducted using the R Packages DPLYR and Tidyverse[44,45]. All CT data associated with this work is archived and freely available for download at Zenodo at the following https://doi.org/10.5281/zenodo.5753695”

In Peredo et al.’s Results section, there is one paragraph (reproduced below) that provides a few clues regarding what they measured and counted on the palates of artiodactyl specimens that they examined. “Minor palatal foramina” are defined as those <1mm, and “major palatal foramina” (MPF) are defined as >1mm in diameter but were reported by Peredo et al. as being as small as 1.0mm. It therefore seems that Peredo et al. regarded MPF as foramina that are ≥1 mm. However, a second, more enigmatic ambiguity in Peredo et al.’s quantification of MPF counts centers on which bones on the palate were surveyed. There is no information in their Methods section or in online supplemental materials that details whether MPF were counted just on the maxilla or on all bones of the palate. In the one relevant section of their Results, Peredo et al. noted that, “... lateral palatal foramina are observed medial to the tooth row…” and that, “lateral palatal foramina are observed in the inter-alveolar septae, the alveolar

margins, and even within the alveoli of missing teeth." These two statements suggest that Peredo et al. counted MPF medial to the toothrow, right at the base of teeth, between teeth, and even within tooth sockets (alveoli) but did not specify what regions of the palate and which bones were surveyed for foramina. It is therefore unclear whether Peredo et al. restricted their counts to the maxilla or whether they also counted MPF on the palatine and premaxilla, which also contribute to the palate in various artiodactyl species. For example, as we noted for the 'archaeocetes' *Basilotritus wardii* and *Dorudon atrox* (above), Peredo et al. identified foramina in the premaxilla as "LPF" (e.g., fig. 4b of [1]), and for *Sus scrofa*, Peredo et al. described a "LPF" that is posterior to the greater palatine foramen on the palatine bone (Figure S2g–i). It is not clear whether these were included in their MPF (or TPF) counts. Given the limited information on methodology in [1], we conclude that it is impossible to determine which MPF were counted.

> "Given the homology of these structures, we sought to document the prevalence of lateral palatal foramina in terrestrial artiodactyls. We observed 81 specimens spanning 61 species of terrestrial artiodactyls and report the presence of lateral palatal foramina in all of them (Table S1). The morphological diversity and number of foramina varies widely: most specimens exhibit one or more clusters of minor palatal foramina (diameter < 1 mm) and at least one major palatal foramen (diameter > 1 mm). We also report the total number and the number of major palatal foramina, as well as the diameter of the major palatal foramina (Table S3). The majority of terrestrial artiodactyls in our dataset preserve between 4 and 8 major lateral palatal foramina (diameter > 1 mm), ranging in size from 1 mm (numerous taxa) to 8.2 mm (*Hippopotamus amphibius*). In each case, the lateral palatal foramina are observed medial to the tooth row (when teeth are present) and are oriented either anteriorly or anterolaterally (Fig. 4); the position and configuration of this morphology in terrestrial artiodactyls is consistent with that observed in aetiocetids and other stem mysticetes[10,20]. In many cases, the lateral palatal foramina are observed in the inter-alveolar septae, the alveolar margins, and even within the alveoli of missing teeth." (p. 3, 2nd paragraph in [1])

In the two brief paragraphs quoted above and in their online supplemental materials, Peredo et al. also provided insufficient information on how "total number of clusters of palatal foramina" (TPF) were quantified. Counts of TPF are shown in their table S2 for all specimens that were examined, and TPF also were plotted in Peredo et al.'s fig. S1, but it is unclear how "clusters" of minor and major foramina were identified/differentiated for any particular specimen. Based on Peredo et al.'s text, we contend that it is impossible to replicate their TPF results. Aside from not defining what a "cluster" of foramina is, Peredo et al. again did not specify whether they

counted clusters on all bones that comprise the palate (palatine, maxilla, premaxilla) or just on the maxilla where LPF of mysticetes are found exclusively [2–5]. Multiple emails with Peredo et al. unfortunately did not help clarify their methods. Therefore we conclude that their TPF data are not interpretable or repeatable, and do not consider them further here.

In our inspection of artiodactyl specimens (Figure 3; Table S2), we commonly observed spongy, cancellous bone with scattered pores, miniscule divots, and tiny holes (typically <0.5mm) at the base of and between cheek teeth that were interspersed with generally small foramina. Such features are not consistent among different specimens of the same species in terms of anatomical location, presence versus absence, ontogenetic stage, sex, and geographic locale (Figures S4–S5). For example, in Figure S5 a small foramen can be seen near the interdental septum between P4 and M1 on the left side of the maxilla in one specimen of *Gazella gazella* (Figure S5c, MSU 7976), but a corresponding foramen does not occur in a similar position in MSU 4596 (Figure S5b) despite being from the same species. The same can be seen in Figure S5e, where a small foramen is observed on the right side of the maxilla near the base of M1 in a specimen of *Gazella leptoceros* (UMMZ 170490) but is absent from that position in another specimen of the same species (Figure S5d; UMMZ 170488). We contend that most of these indentations and openings at the base of and between teeth characterize resorption and remodeling of alveolar bone. Indeed, the bone surrounding the maxillary dentition is porous in some specimens (Figures S2a–b,g–h, S4f–h), but not in others (Figures S4e, j, S5a–c). Similar features of bone quality and texture in the interdental septa and surrounding alveolar bone have been used as an identifier of periodontal disease in ancient human populations, as well as in other mammals [17–20]. This is likely the case in terrestrial artiodactyl specimens with these tiny foramina and regions of increased porosity as they are generally not bilaterally symmetrical across the palate and are not positionally consistent across specimens of the same species (if present at all). This indicates that these features relate to individual life histories rather than consistent morphology inherited through descent. We commonly observed extensively pitted bone with larger holes at the base of cheek teeth in many skulls from zoo animals, which we attribute to inadequate/inappropriate diet, environmental conditions, periodontal disease, or other pathologies related to life in captivity. Such features were notably less common in specimens collected from the wild. Given these observations, with few exceptions, we avoided specimens from zoological parks in our study. For the reasons discussed above, we do not consider such features, which are nearly always <1mm in diameter, to be LPF (or in some cases even foramina).

By contrast, in extant mysticetes (Figure 3b–d), LPF are generally large (>1mm), commonly have long sulci, and are arrayed on the maxilla in a bilaterally symmetrical pattern that is broadly consistent among different individuals of the same species. In the best characterized aetiocetid

toothed mysticete (*Aetiocetus weltoni*, Figure S6), the 15 LPF are ~1mm in diameter, generally have anterolaterally directed sulci, are distributed in a broadly bilaterally symmetrical pattern on the maxillae (7 on right side of palate, 8 on left side), are medial to the toothrow, and connect to the SAC and the IOC as in extant mysticetes [3–5]. We therefore contend that MPF should be counted only on the maxilla where LPF are known to be found found in both extant and extinct mysticetes, and associated sulci should be noted, as well as the distribution/pattern of foramina on left and right sides of the palate. We reiterate that Peredo et al. did not specify which bones were surveyed for MPF in [1]. Peredo et al. considered foramina on the premaxilla to be "LPF" even though they are not homologous with true LPF that are only found on the maxilla. Furthermore they may have counted MPF on the palatine (unclear from Peredo et al.'s published text), and even considered holes within dental alveoli to be "LPF", all of which might explain their higher counts of MPF in terrestrial artiodactyl species relative to our data (Figure 3e–f; Table S2). We could not, however, arrive at a plausible explanation for the consistent and often extreme undercounting of MPF in mysticete specimens (Figure 3b–f; Table S2). Furthermore, Peredo et al. did not systematically count and measure sulci associated with MPF or minor palatal foramina. Sulci are prominent and consistent features of LPF anatomy in Mysticeti (Figures 3b–d, S6; [3–5]). Sulci should, therefore, be characterized when analyzing potential "LPF" in taxa that are distantly related to Mysticeti.

Our issues with MPF counts and lack of observations of sulci, as described above, are best highlighted by direct comparison of two specimens used in [1]: the terrestrial artiodactyl *Bubalus bubalis* (water buffalo) and the mysticete *Eschrichtius robustus* (gray whale). Peredo et al. counted more large foramina on the smooth palate of *B. bubalis* (7 MPF; UMMZ 156568) than on the palate of *E. robustus* (5 MPF; USNM 364973) even though the latter is riddled with a multitude of the large LPF and associated sulci typically seen in baleen-bearing mysticetes (Figure 3a vs Figure 3c–d; also see Figure S6c–d). The only large holes (≥1mm) that we observed in the same water buffalo specimen examined by Peredo et al. are on the palatines. No sulci are evident on the maxilla, except for the shallow grooves that extend from the two greater palatine foramina at the maxillopalatine suture (Figure 3a). Peredo et al. reported MPF of 1 mm, 1.4 mm, 1.5 mm, 1.5 mm, 1.6 mm, 1.9 mm, 2.0 mm, and 2.4 mm in diameter for this specimen of *B. bubalis* (table S3 in [1]). We could not discern any foramina with these dimensions on the maxilla (Figure 3a). We examined an additional specimen of *B. bubalis* (UMMZ 156569) and counted just one MPF on the maxilla rather than the eight reported by [1] for the same specimen. By contrast, the inset focused on just a small region of the left maxilla in *E. robustus* (Figure 3d) clearly shows >15 large foramina, and we recorded a total of 57 MPF on the maxilla of this specimen—more than an order of magnitude greater than Peredo et al.'s count of 5 (Figure 3c–). This discrepancy could be explained by a mix-up of USNM specimen numbers, in which Peredo et al. counted MPF on a gray whale with much fewer LPF but then

misreported the specimen ID as USNM 364973. However, we completed preliminary maxillary MPF counts on seven additional gray whale skulls at USNM, and MPF ranged from 29 to 70 (median 58; mean 55.1).

Overall, MPF counts in [1] are higher than ours for terrestrial artiodactyls with few exceptions, and their MPF counts for mysticetes are much lower than ours for all four baleen-whale specimens in their dataset (Figure 3e–f). As further examples, all four specimens of terrestrial artiodactyl that we reanalyzed from [1] had substantially lower counts of MPF than what were reported in [1]. These include *Aepyceros melampus* (UMMZ 124571; 8 MPF reported in [1]), *Antilocapra americana* (UMMZ 65502; 10 MPF in [1]), *Bubalus bubalis* (UMMZ 156568; 7 MPF in [1]), and *Damaliscus pygargus* (UMMZ 167702; 6 MPF in [1]; Figure S4a), all of which were found to have zero MPF on the maxilla upon reanalysis. Cursory inspection of numerous other terrestrial artiodactyl specimens reported by [1] from UMMZ suggests that many specimens analyzed by Peredo et al. were raised in captivity in the midwestern U.S., possessed dental pathologies, and/or were not adult individuals, which may be problematic (see above). Regardless, our counts of MPF on terrestrial artiodactyls are extremely low, even on specimens utilized in [1], and in many cases we were not able to discern foramina corresponding to the diameters reported by [1].

Systematic differences in MPF counts (i.e., consistent overcounts in terrestrial artiodactyls and undercounts in mysticetes) uniformly favor Peredo et al.'s primary thesis that "LPF" are widespread across Artiodactyla, that they are just as abundant in terrestrial artiodactyls as in mysticetes, and that inference of baleen in extinct taxa based on the presence of "LPF" is therefore problematic. However, our reanalysis of the same specimens used in [1], as well as study of additional specimens at AMNH and USNM, refutes Peredo et al.'s hypothesis of parity in MPF counts across Artiodactyla. Coupled with our reanalysis of Peredo et al.'s CT data, current evidence suggests that LPF only occur in mysticetes.

It is important to stress again that the maxillary MPF of various terrestrial artiodactyl species are not even structural homologs to maxillary MPF in mysticetes. In contrast to Peredo et al.'s assertion that all palatal foramina in Artiodactyla are "LPF" and therefore homologous across divergent subclades [1], our reanalysis of CT scans showed that the "internal plumbing" of maxillary foramina in terrestrial artiodactyls (connecting to a PTC that has no direct connections to the cheek teeth; Figures 1–2, S1–S2) is not consistent with that of LPF in mysticetes (connecting to the IOC and SAC [2–3]). Therefore, counts of MPF do not represent the same anatomical structures in different taxa and the statistical tests in which Peredo et al. compared MPF counts across divergent groups are thus not valid. In our current study, we used newly generated MPF data to test Peredo et al.'s assertion that MPF counts are not significantly

different (Figure 3e–f) using the same statistical test that they employed (Tukey's Honest Significant Difference test). However, we stress that this test has two shortcomings when applied in this context: 1) comparing counts of completely different anatomical structures in different taxonomic groups is not a sound approach, and 2) the test does not account for phylogenetic structure (i.e., each species is treated as an independent data point). A proper statistical test would compare only homologous foramina/canals to each other and account for statistical dependence due to shared phylogenetic history. Additional careful analyses of CT data are required to track the rostral canals of palatal foramina across Artiodactyla before valid evolutionary mapping and statistical tests can be applied (Ekdale et al., in prep.).

Methods for counting MPF utilized in the current study

Explicit and clearly-presented methods are key to any scientific endeavor, so we detail our methodology for counting MPF and characterizing associated sulci (below). We generally restricted analyses to specimens of wild-caught adults (M3 erupted) with undamaged palates. Exceptions to this were for specimens reported in [1] that were reanalyzed here. *Antilocapra americana* (UMMZ 65502) was a captive-raised specimen that was missing portions of the premaxillae and had severe dental pathologies including in vivo loss of the left first molar and decalcification of the maxillae, producing windows that laterally exposed tooth roots. The palate was highly pitted with extensive remodeling of the alveolar bone. We note that multiple other specimens of this species are held within the collection at UMMZ, and this is the only individual with this degree of pathology. *Damaliscus pygargus* (UMMZ 167702) had partially erupted third molars and was raised at the Detroit Zoo in Michigan, USA. As with the specimen of *A. americana*, this specimen displayed remodeling of the alveolar bone, perhaps due to an inadequate diet.

Sixty-one terrestrial artiodactyl species and 29 odontocete species were scored from specimens at **AMNH** (American Museum of Natural History, New York, New York) and **UMMZ** (University of Michigan Museum of Zoology, Ann Arbor, Michigan). Ten mysticete species were scored from specimens at **USNM** (National Museum of Natural History, Smithsonian Institution, Washington, District of Columbia) (Table S2). All specimens of extant mysticetes reported in [1] were reanalyzed, as well as four specimens of terrestrial artiodactyl (Figure 3, Table S2). We examined high resolution photographs (provided by P. Myers) of bovid specimens at **UMMZ** and **MSU** (Michigan State University, Lansing, Michigan) used by [1], as well as other specimens that Peredo et al. did not examine.

MPF-pal: Total count of all major palatal foramina occurring within the palatine or at the suture between the palatine and maxilla. Palatal foramina must be ≥1.0 mm in greatest diameter measured parallel to the palate surface in order to be counted as major palatal foramina. Only

foramina occurring on the same plane as the palate are to be counted. Foramina that open at the maxillopalatine suture are counted under MPF-pal rather than MPF-max, as these are generally the greater palatine foramina that connect internally to the palatine canal (e.g., Figure 3a).

MPF-max: Total count of all major palatal foramina occurring within the maxilla and medial to the tooth row (for taxa with teeth). Palatal foramina must be ≥1.0 mm in greatest diameter measured parallel to the palate surface in order to be counted as major palatal foramina. Only foramina occurring on the same plane as the palate are to be counted.

MPF-pmx: Total count of all major palatal foramina occurring within the premaxilla and medial to the tooth row (for taxa with teeth). Palatal foramina must be ≥1.0 mm in greatest diameter measured parallel to the palate surface in order to be counted as major palatal foramina. Only foramina occurring on the same plane as the palate are to be counted. We did not include incisive foramina, which are large openings associated with the vomeronasal organ, in our MPF-pmx counts.

Sulci-pal: Total count of all sulci associated with foramina (of any size) within the palatine or at the suture between the palatine and maxilla. Sulci must be ≥5.0 mm in greatest straight-line length measured parallel to the palate surface in order to be counted. Only sulci occurring on the same plane as the palate are to be counted. The location designation of the sulcus (e.g., sulci-pal vs. sulci-max vs. sulci-pmx) will be determined based on the location of the foramen from which the sulcus originates (see descriptions for MPF-pal, MPF-max, and MPF-pmx).

Sulci-max: Total count of all sulci associated with foramina (of any size) within the maxilla and medial to the tooth row (for taxa with teeth). Sulci must be ≥5.0 mm in greatest straight-line length measured parallel to the palate surface in order to be counted. Only sulci occurring on the same plane as the palate are to be counted. The location designation of the sulcus (e.g., sulci-pal vs. sulci-max vs. sulci-pmx) will be determined based on the location of the foramen from which the sulcus originates (see descriptions for MPF-pal, MPF-max, and MPF-pmx).

Sulci-pmx: Total count of all sulci associated with foramina (of any size) within the premaxilla and medial to the tooth row (for taxa with teeth). Sulci must be ≥5.0 mm in greatest straight-line length measured parallel to the palate surface in order to be counted. Only sulci occurring on the same plane as the palate are to be counted. The location designation of the sulcus (e.g., sulci-pal vs. sulci-max vs. sulci-pmx) will be determined based on the location of the foramen from which the sulcus originates (see descriptions for MPF-pal, MPF-max, and MPF-pmx).

Sulci - greatest length (mm): Measurement of the longest sulcus observed on the palate (regardless of location). To be measured, sulcus must be ≥5.0 mm in greatest length. If the longest sulcus is <5.0 mm, then no measurement will be made. Length will be measured from the opening of the foramen (the leading edge) onto the palate surface to the furthest discernible point away from the foramen that the sulcus can be noted by eye. Sulcus length will be measured parallel to the palate surface along a straight-line length. If the palate is anteroposteriorly arched (e.g., as in balaenid mysticetes), then a curved-line length following the palate should be taken. Only sulci occurring on the same plane as the palate are to be counted.

Sulci - greatest length, max (mm): Measurement of the longest sulcus observed from a foramen originating on the maxilla. To be measured, sulcus must be ≥5.0 mm in greatest length. If the longest sulcus is <5.0 mm, then no measurement will be made. Length will be measured from the opening of the foramen (the leading edge) onto the palate surface to the furthest discernible point away from the foramen that the sulcus can be noted by eye. Sulcus length will be measured parallel to the palate surface along a straight-line length. If the palate is anteroposteriorly arched, then a curved-line length following the palate should be taken. Only sulci occurring on the same plane as the palate are to be counted.

Sulci - greatest length, location: Description of where the longest sulcus occurs on the palate. The location designation of the sulcus (e.g., sulci-pal vs. sulci-max vs. sulci-pmx) will be determined based on the location of the foramen from which the sulcus originates (see descriptions for MPF-pal, MPF-max, and MPF-pmx). If all sulci on the palate are <5.0 mm in greatest length, then no sulci will be measured or noted here. Descriptive notes on other large or notable sulci are also appropriate here.

Sulci - orientation: Description of the general arrangement and orientation of the sulci on the palate. If all sulci on the palate are <5.0 mm in greatest length, then no description of sulci should be noted here.

Supplemental References

(References cited in Supplemental Materials that do not appear in main text of the article)

[10] FEI Visualization Sciences Group. Avizo 3D 2021.2. Berlin: Konrad-Zuse-Zentrum für Informationstechnik (2021).

[11] Sisson, S. & Grossman, J. D. *The Anatomy Of The Domestic Animals*, 3rd edn. Philadelphia, PA: W. B. Saunders Company (1943).

[12] O'Brien, H. D. Cranial arterial patterns of the alpaca (Camelidae: *Vicugna pacos*). *R. Soc. Open Sci.* **4,** 160967; 10.1098/rsos.160967 (2017).
[13] O'Brien, H. D., Gignac, P. M., Heironymus, T. L., & Witmer, L. A. A comparison of postnatal arterial patterns in a growth series of giraffe (Artiodactyla: *Giraffa camelopardalis*). *PeerJ* **4,** e1696; 10.7717/peerj.1696 (2016).
[14] Fordyce, R. E. 2002. *Simocetus rayi* (Odontoceti, Simocetidae, new family): a bizarre new archaic Oligocene dolphin from the eastern North Pacific. *Smithson. Contrib. Paleobiol.* **93,** 185–222 (2002).
[15] Uhen, M. D. New species of protocetid archaeocete whale, *Eocetus wardii* (Mammalia: Cetacea) from the middle Eocene of North Carolina. *J Paleontol.* **73,** 512–528; 10.1017/S002233600002802X (1999).
[16] Boessenecker, R.W., Beatty, B.L., & Geisler, J.H. New specimens and species of the Oligocene toothed baleen whale *Coronodon* from South Carolina and the origin of Neoceti. *PeerJ* **11**, e14795; 10.7717/peerj.14795 (2023).
[17] Kerr, N.W. A method of assessing periodontal status in archaeologically derived skeletal material. *J. Paleopathol.* **2,** 67–78 (1988).
[18] Bertl, K., Tangl, S., Rybaczek, T., Berger, B., Traindl-Prohazka, M., Schuller-Götzburg, P., & Grossschmidt, K. Prevalence and severity of periodontal disease in a historical Austrian population. *J. Periodontal Res.* **55**(6), 931–945 (2020).
[19] Vilà, C., Urios, V., & Castroviejo, J. Tooth losses and anomalies in the wolf (*Canis lupus*). *J. of Zool.* **71,** 968–971 (1993).
[20] Loch, C., Simões-Loes, P.C., & Drehmer, C.J. Numerical anomalies in the dentition of southern fur seals and sea lions (Pinnipedia: Otariidae). *Zoologia* **27**(3), 477–482 (2010).

SUPPLEMENTAL FIGURE LEGENDS

Figure S1. Additional digital rendering of skull and segmentation of rostral canals of *Hippopotamus amphibius* in multiple views. Dorsal view (**a–c**) with skull rendered semi-transparent to reveal digital segmentation of internal canals (**a**), segmentations of canals and teeth with cranium removed (**b**), and segmentations of canals with teeth and cranium removed (**c**). Medial view (**d–f**) with skull rendered semi-transparent to reveal digital segmentation of internal canals (**d**), segmentations of canals and teeth with cranium removed (**e**), and segmentations of canals with teeth and cranium removed (**f**). Lateral view (**g–i**) with skull rendered semi-transparent to reveal digital segmentation of internal canals (**g**), segmentations of canals and teeth with cranium removed (**h**), and segmentations of canals with teeth and cranium removed (**i**). Anterior view (**j–l**) with skull rendered semi-transparent to reveal digital segmentation of internal canals (**j**), segmentations of canals and teeth with cranium removed (**k**), and segmentations of canals with teeth and cranium removed (**l**). Note that there are no

direct connections between the PTC and cheek teeth, suggesting that this canal is not the SAC. Abbreviations: **dC** - deciduous canine; **dI1** - deciduous first incisor; **dI2** - deciduous second incisor; **dP2** - deciduous second premolar; **dP3** - deciduous third premolar; **dP4** - deciduous fourth premolar; **GPF** - greater palatine foramen; **IOC** - infraorbital canal; **IOF** - infraorbital foramen; **PMC** - premaxillary canal; **PTC** - palatine canal (identified as SAC in [1]); **PTF** - palatine foramen (identified as LPF in [1]); **SAC** - superior alveolar canal.

Figure S2. CT slices through rostrum of terrestrial artiodactyls. CT slices through rostrum of *Lama glama* (TMM M-2052) (**a–c**), *Odocoileus virginianus* (USNM VZ 118627) (**d–f**), *Sus scrofa* (USNM VZ 260907) (**g–i**), and *Tayassu pecari* (USNM 406851) (**j–l**). Coronal slices of *L. glama* (**a**) and *O. virginianus* (**d**) and oblique slice of *S. scrofa* (**g**) approximate the locations of slices depicted in fig. 2 of [1]. Coronal slice for *T. pecrari* (**j**) in comparable position to other coronal/oblique slices. Parasagittal slices cut through either IOC (**b,e,h,k**) or PTC (**c,f,i,l**). Dashed lines in parasagittal slices represent coronal/oblique slices depicted in (**a,d,g,j**). Abbreviations: **GPF** - greater palatine foramen; **IOC** - infraorbital canal; **PNS** - pneumatic space (identified as SAC in [1]); **PTC** - palatine canal (identified as SAC in [1]); **PTF** - palatine foramen (identified as LPF in [1]); **SAC** - superior alveolar canal. Each scale bar is 2cm.

Figure S3. Comparison of 3D renderings of rostral canals of *Aetiocetus weltoni* (UCMP 122900). (**a**) Original image from fig. 2b of [3] compared to (**b**) modified image in fig. 5d of [1]. Enlargements showing regions of original image (**c**) that differ in [1]. (**d**). Abbreviations: **LPC** - lateral palatal canals leading to LPF; **LPF** - lateral palatal foramina. Images from [1,3] licensed under a Creative Commons Attribution 4.0 International License (http://creativecommons.org/licenses/by/4.0/). Anatomical labels in (**a–b**) reformatted to conform with figures of the current study, but identifications of structures are not changed. Pointers crossing white space removed in (**c–d**).

Figure S4. Left half of the palate of terrestrial artiodactyl specimens used in [1] showing the condition of maxilla and palatine, along with Peredo et al.'s MPF counts (red text). (**a**) *Damaliscus pygargus* (UMMZ 166702), (**b**) *Damaliscus lunatus* (UMMZ 124070), (**c**) *Boselaphus tragocamelus* (UMMZ 167639), (**d**) *Bos taurus* (UMMZ 86878), (**e**) *Ammotragus lervia* (UMMZ 97068), (**f**) *Ovis canadensis* (UMMZ 102446), (**g**) *Oryx gazella* (UMMZ 168361), (**h**) *Oreamnos americanus* (UMMZ 60546), (**i**) *Gazella leptoceros* (UMMZ 170490), and (**j**) *Eudorcas thomsonii* (UMMZ 124084). Note that foramina on maxilla, if present, primarily are tiny (<0.5mm), within the interdental septa, within alveoli (e.g., panel **d**), and/or at the base of teeth, rather than more medial to the palatal margin, as in mysticetes. The large opening in maxilla of (**a**) is postmortem damage, not an anatomical feature. Abbreviations: **GPF** - greater palatine

foramen; **MPF** - total number of major palatal foramina (≥ 1mm diameter); **MX** - maxilla; **PA** - palatine. Each scale bar is 1cm with mm increments. Photos provided by P. Myers.

Figure S5. Palates of *Gazella* spp. showing the condition of premaxilla, maxilla, and palatine. (**a**) *G. dorcas* (UMMZ 161037), (**b**) *G. gazella* (MSU 4596), (**c**) *G. gazella* (MSU 7976), (**d**) *G. leptoceros* (UMMZ 170488), (**e**) *G. leptoceros* (UMMZ 170490), and (**f**) *G. subgutturosa* (UMMZ 170470). MPF counts (red) of Peredo et al. are shown for single specimen used in [1] (**e**). Note that palatal foramina on the maxilla are neither widespread nor consistent intragenerically or intraspecifically. When observed, foramina on maxilla do not occur on both sides of the palate, contrary to the condition seen in the LPF of mysticetes (Figure 3b–d). Miniscule foramina (<0.5mm) on maxilla primarily are observed in the interdental septum and/or at the base of teeth, rather than more medial to the palatal margin, as in mysticetes. Abbreviations: **GPF** - greater palatine foramen; **LPF** - lateral palatal foramina; **MPF** - total number of major palatal foramina (≥ 1mm diameter); **MX** - maxilla; **PA** - palatine. Each scale bar is 1cm with mm increments. Photos provided by P. Myers.

Figure S6. Palates of cetaceans showing condition of maxilla. Left half of palate with inset close-ups of the extant mysticetes (**a**–**b**) *Balaenoptera borealis* (USNM 593415) and (**c**–**d**) *Eschrichtius robustus* (USNM 364973); the extinct toothed-mysticete (**e**–**f**) *Aetiocetus weltoni* (UCMP 122900); and the extant odontocete (**g**–**h**) *Tursiops truncatus* (USNM 550969). LPF are marked with a red star. Note that each mysticete (**a–f**) has many LPF, often associated with elongate sulci, and there are none on the palate of *T. truncatus*. Specimens of extant cetaceans (**a–d,g–h**) included in [1]. Line drawing of *A. weltoni* (**e**) redrawn after [4] to better show the arrangement of LPF on the palate. Scale bars are 10cm for extant mysticetes (**b,d**) and 3cm with mm increments for *A. weltoni* (**f**).

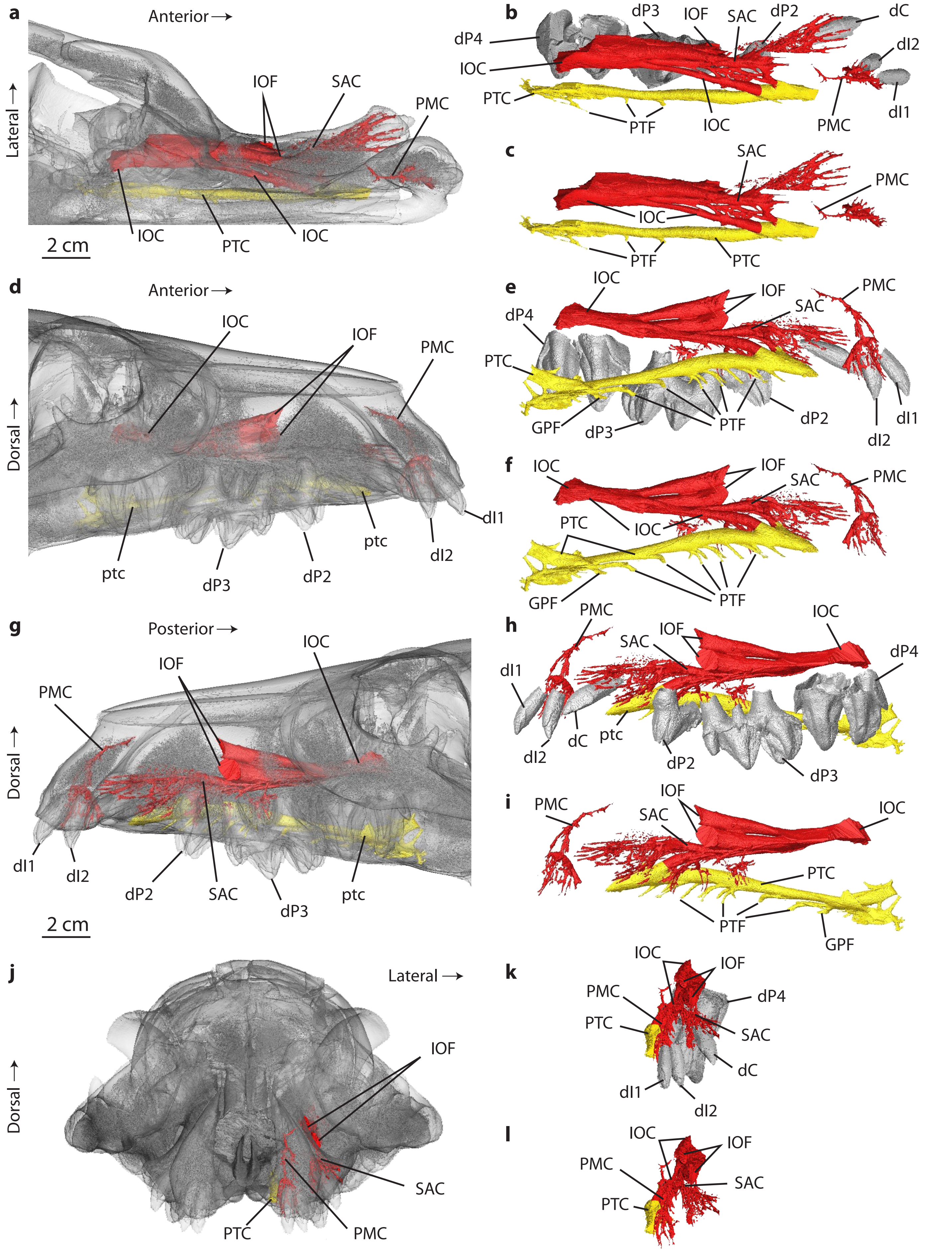

Figure S1. Additional digital rendering of skull and segmentation of rostral canals of *Hippopotamus amphibius* in multiple views. Dorsal view (**a–c**) with skull rendered semi- transparent to reveal digital segmentation of internal canals (**a**), segmentations of canals and teeth with cranium removed (**b**), and segmentations of canals with teeth and cranium removed (**c**). Medial view (**d–f**) with skull rendered semi-transparent to reveal digital segmentation of internal canals (**d**), segmentations of canals and teeth with cranium removed (**e**), and segmentations of canals with teeth and cranium removed (**f**). Lateral view (**g–i**) with skull rendered semi-transparent to reveal digital segmentation of internal canals (**g**), segmentations of canals and teeth with cranium removed (**h**), and segmentations of canals with teeth and cranium removed (**i**). Anterior view (**j–l**) with skull rendered semi-transparent to reveal digital segmentation of internal canals (**j**), segmentations of canals and teeth with cranium removed (**k**), and segmentations of canals with teeth and cranium removed (**l**). Note that there are no direct connections between the PTC and cheek teeth, suggesting that this canal is not the SAC. Abbreviations: **dC** - deciduous canine; **dI1** - deciduous first incisor; **dI2** - deciduous second incisor; **dP2** - deciduous second premolar; **dP3** - deciduous third premolar; **dP4** - deciduous fourth premolar; **GPF** - greater palatine foramen; **IOC** - infraorbital canal; **IOF** - infraorbital foramen; **PMC** - premaxillary canal; **PTC** - palatine canal (identified as SAC in [1]); **PTF** - palatine foramen (identified as LPF in [1]); **SAC** - superior alveolar canal.

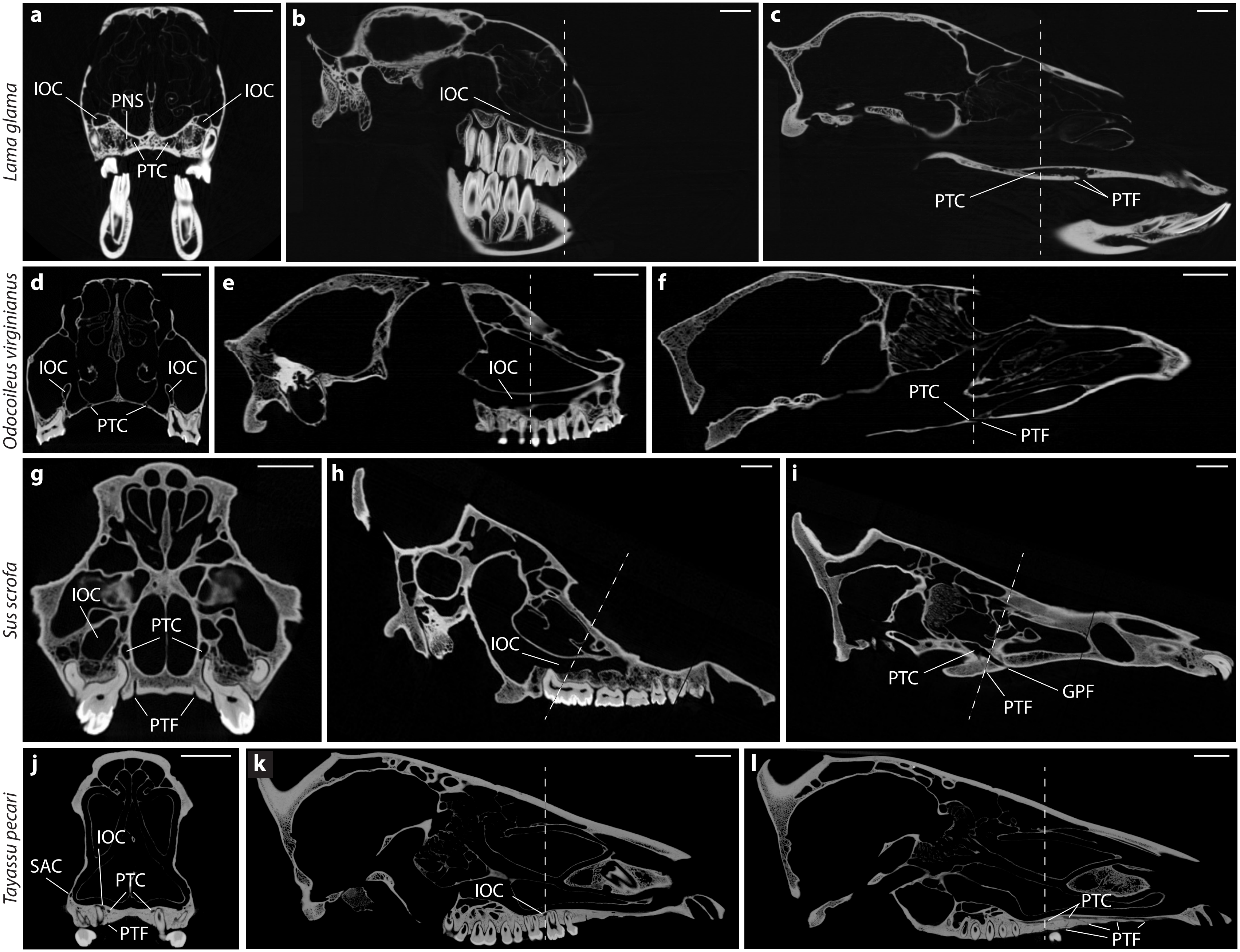

Figure S2. CT slices through rostrum of terrestrial artiodactyls. CT slices through rostrum of *Lama glama* (TMM M-2052) (**a–c**), *Odocoileus virginianus* (USNM VZ 118627) (**d–f**), *Sus scrofa* (USNM VZ 260907) (**g–i**), and *Tayassu pecari* (USNM 406851) (**j–l**). Coronal slices of *L. glama* (**a**) and *O. virginianus* (**d**) and oblique slice of *S. scrofa* (**g**) approximate the locations of slices depicted in fig. 2 of [1]. Coronal slice for *T. pecrari* (**j**) in comparable position to other coronal/oblique slices. Parasagittal slices cut through either IOC (**b,e,h,k**) or PTC (**c,f,i,l**). Dashed lines in parasagittal slices represent coronal/oblique slices depicted in (**a,d,g,j**). Abbreviations: **GPF** - greater palatine foramen; **IOC** - infraorbital canal; **PNS** - pneumatic space (identified as SAC in [1]); **PTC** - palatine canal (identified as SAC in [1]); **PTF** - palatine foramen (identified as LPF in [1]); **SAC** - superior alveolar canal. Each scale bar is 2cm.

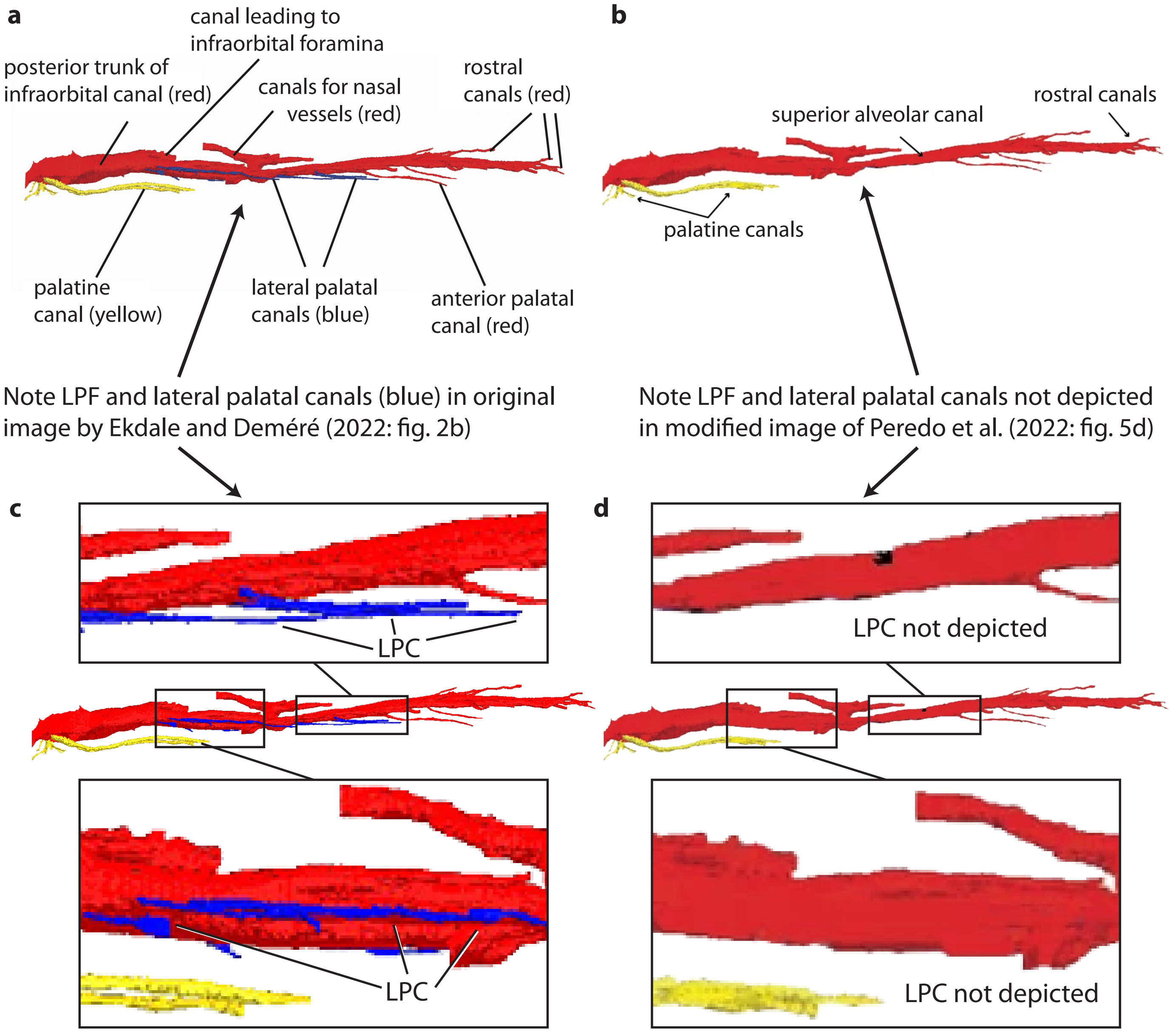

Figure S3. Comparison of 3D renderings of rostral canals of *Aetiocetus weltoni* (UCMP 122900). (**a**) Original image from fig. 2b of [3] compared to (**b**) modified image in fig. 5d of [1]. Enlargements showing regions of original image (**c**) that differ in [1]. (**d**). Abbreviations: **LPC** - lateral palatal canals leading to LPF; **LPF** - lateral palatal foramina. Images from [1,3] licensed under a Creative Commons Attribution 4.0 International License (http://creativecommons.org/licenses/by/4.0/). Anatomical labels in (**a–b**) reformatted to conform with figures of the current study, but identifications of structures are not changed. Pointers crossing white space removed in (**c–d**).

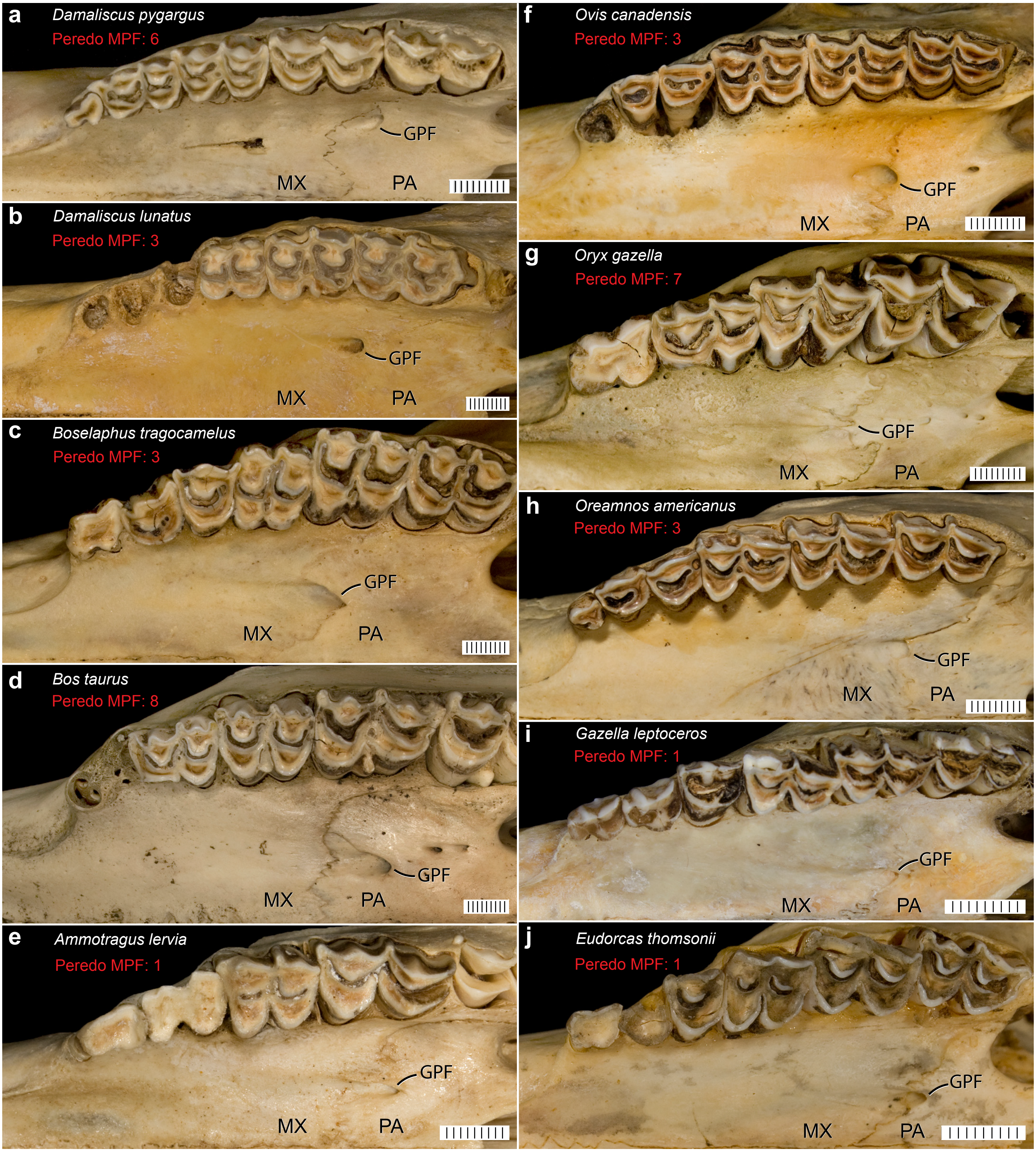

Figure S4. Left half of the palate of terrestrial artiodactyl specimens used in [1] showing the condition of maxilla and palatine, along with Peredo et al.'s MPF counts (red text). (**a**) *Damaliscus pygargus* (UMMZ 166702), (**b**) *Damaliscus lunatus* (UMMZ 124070), (**c**) *Boselaphus tragocamelus* (UMMZ 167639), (**d**) *Bos taurus* (UMMZ 86878), (**e**) *Ammotragus lervia* (UMMZ 97068), (**f**) *Ovis canadensis* (UMMZ 102446), (**g**) *Oryx gazella* (UMMZ 168361), (**h**) *Oreamnos americanus* (UMMZ 60546), (**i**) *Gazella leptoceros* (UMMZ 170490), and (**j**) *Eudorcas thomsonii* (UMMZ 124084). Note that foramina on maxilla, if present, primarily are within the interdental septa, within alveoli (e.g., panel **d**), and/or at the base of teeth, rather than more medial to the palatal margin, as in mysticetes. The large opening in maxilla of (**a**) is postmortem damage, not an anatomical feature. Abbreviations: **GPF** - greater palatine foramen; **MPF** - total number of major palatal foramina (≥ 1mm diameter); **MX** - maxilla; **PA** - palatine. Each scale bar is 1cm with mm increments. Photos provided by P. Myers.

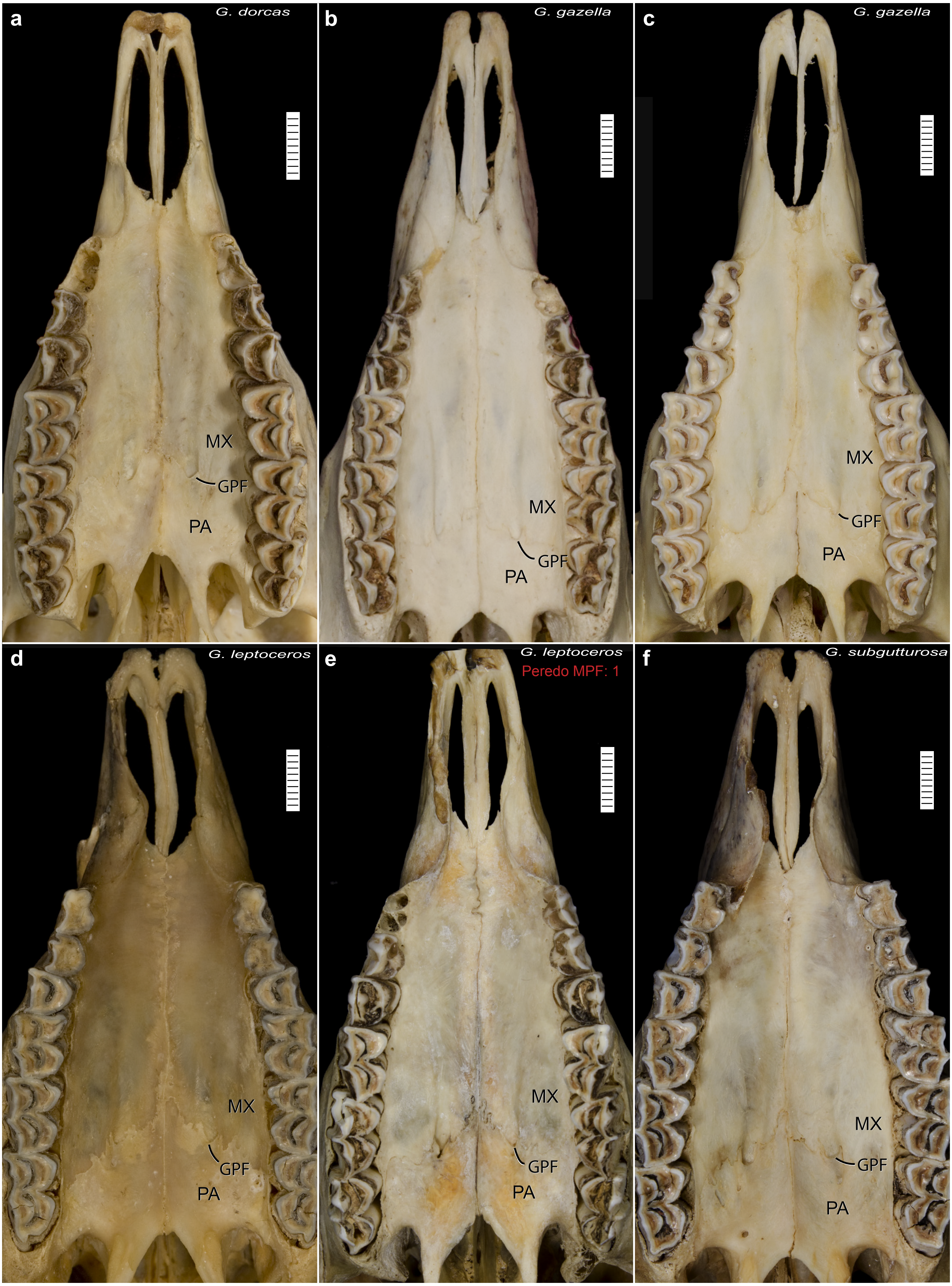

Figure S5. Palates of *Gazella* spp. showing the condition of premaxilla, maxilla, and palatine. (**a**) *G. dorcas* (UMMZ 161037), (**b**) *G. gazella* (MSU 4596), (**c**) *G. gazella* (MSU 7976), (**d**) *G. leptoceros* (UMMZ 170488), (**e**) *G. leptoceros* (UMMZ 170490), and (**f**) *G. subgutturosa* (UMMZ 170470). MPF counts (red) of Peredo et al. are shown for single specimen used in [1] (**e**). Note that palatal foramina on the maxilla are neither widespread nor consistent intragenerically or intraspecifically. When observed, foramina on maxilla do not occur on both sides of the palate, contrary to the condition seen in the LPF of mysticetes (Figure 3b-d). Miniscule foramina on maxilla primarily are observed in the interdental septum and/or at the base of teeth, rather than more medial to the palatal margin, as in mysticetes. Abbreviations: **GPF** - greater palatine foramen; **LPF** - lateral palatal foramina; **MPF** - total number of major palatal foramina (≥ 1mm diameter); **MX** - maxilla; **PA** - palatine. Each scale bar is 1cm with mm increments. Photos provided by P. Myers.

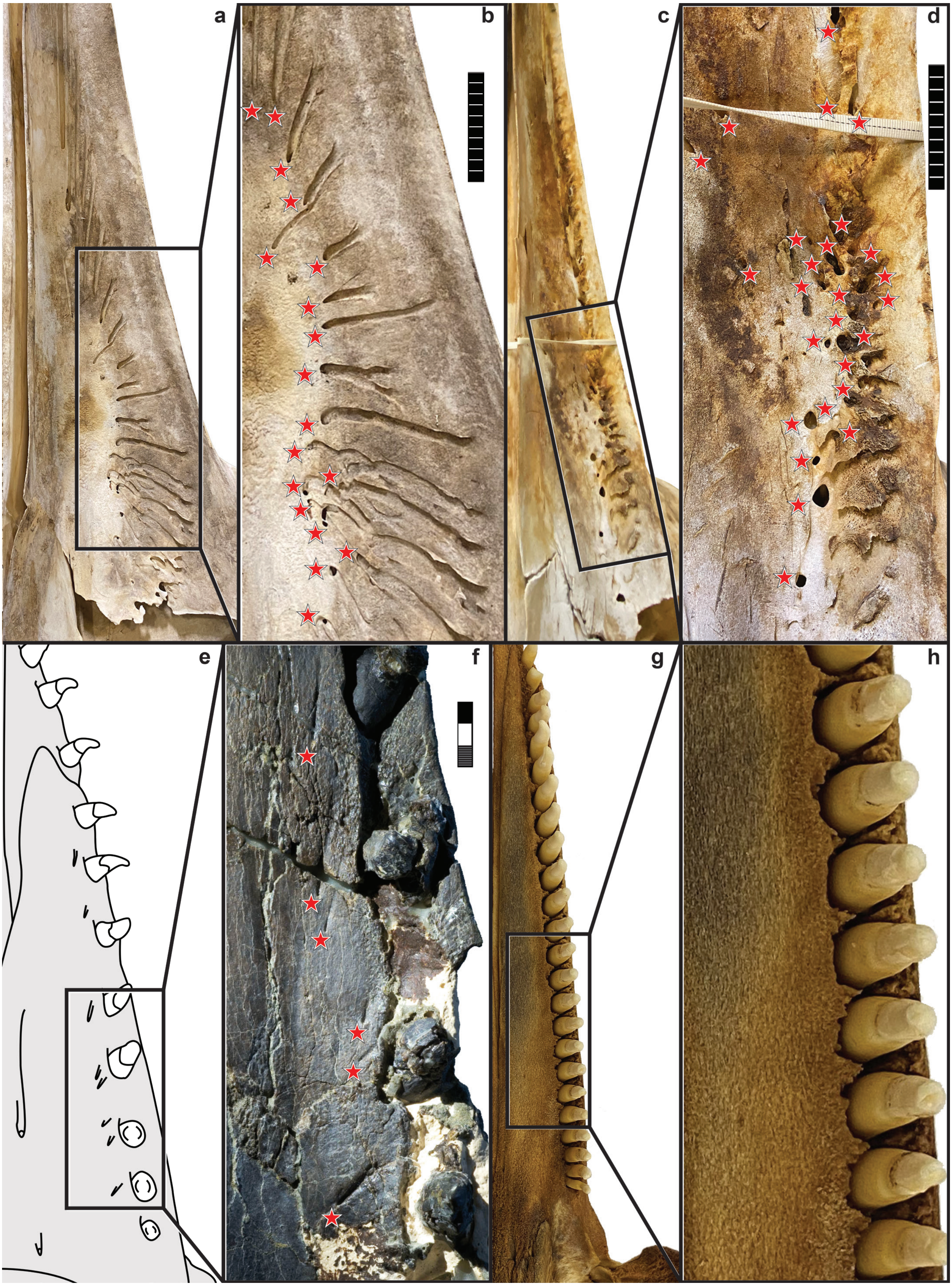

Figure S6. Palates of cetaceans showing condition of maxilla. Left half of palate with inset close-ups of the extant mysticetes (**a-b**) *Balaenoptera borealis* (USNM 593415) and (**c-d**) *Eschrichtius robustus* (USNM 364973); the extinct toothed-mysticete (**e-f**) *Aetiocetus weltoni* (UCMP 122900); and the extant odontocete (**g-h**) *Tursiops truncatus* (USNM 550969). LPF are marked with a red star. Note that each mysticete (**a-f**) has many LPF, often associated with elongate sulci, and there are none on the palate of *T. truncatus*. Specimens of extant cetaceans (**a-d,g-h**) included in [1]. Line drawing of *A. weltoni* (**e**) redrawn after [4] to better show the arrangement of LPF on the palate. Scale bars are 10 cm for extant mysticetes (**b,d**) and 3 cm with mm increments for *A. weltoni* (**f**).

Table S1. Parameters of CT data of terrestrial artiodactyls. Number of slices for datasets of *Odocoileus virginianus* and *Sus scrofa* were overreported by Peredo et al. (table S2 in [1]).

Taxon (specimen number)	Number of slices	Interslice spacing (mm)	Interpixel spacing (mm)	Resolution (pixels)
Hippopotamus amphibius (UMMZ 101782)	1744	0.121616	0.121616	1284x1821
Lama glama (TMM M-2052)	426	0.7	0.1768	1024x1024
Odocoileus virginianus (USNM VZ 118627)	1036	0.3	0.267578	512x512
Sus scrofa (USNM VZ 260907)	1486	0.3	0.421875	512x512
Tayassu pecari (USNM 406851)	1907	0.1441	0.1441	928x1185
